# Supplementary material for: Integrating de novo and inherited variants in 42,607 autism cases identifies mutations in new moderate-risk genes
Source: Nat Genet. 2022 Aug 18;54(9):1305–19. doi: 10.1038/s41588-022-01148-2 (PMC9470534; doi:10.1038/s41588-022-01148-2)
Supplement: Supplementary file 1 — Supplementary Note, Supplementary Table Legends, Supplementary Figs. S1–S24, References. [file 41588_2022_1148_MOESM1_ESM.pdf]

nature portfolio

https://doi.org/10.1038/s41588-022-01148-2

## Supplementary information

# Integrating de novo and inherited variants in 42,607 autism cases identifies mutations in new moderate-risk genes

In the format provided by the authors and unedited

# Contents


**Supplementary Note** 4

***Variant-level quality control*** *4*

Data processing 4

Variant annotations 5

Variant filtering on rare inherited LoFs 6

***Sample-level quality control*** *9*

Relatedness and Ancestry 9

Phenotype 9

***Copy number variants*** *11*

***Other ASD cohorts*** *12*

Simon Simplex Collection (SSC) 12

Autism Sequencing Consortium (ASC) 13

MSSNG 13

**Supplementary Table Legends** 15

**Supplementary Data Legends** 19

**Supplementary Figure S1: Transmission disequilibrium of LoF variants of chromosome X genes** 22

**Supplementary Figure S2: Transmission disequilibrium of deleterious missense (D-mis) variants** 24

**Supplementary Figure S3: Number of genes in each gene set and pairwise overlaps between gene sets before (A) and after (B) excluding known ASD/NDD genes** 27

**Supplementary Figure S4: Inherited LoF variants in genes prioritized by A-risk are not associated with phenotype severity in cases** 29

**Supplementary Figure S5: Comparing carrier rates of LoFs between pseudo-controls and three panels of population-based references for genes selected for replication** 31

**Supplementary Figure S6: Empirical relationship between haploid LoF mutation rate, cumulative allele frequency (CAF) of HC LoFs, and fraction of de novo LoFs in ASD cases** 33

**Supplementary Figure S7: In genes selected for replication, most LoFs are ultra-rare** 35

**Supplementary Figure S8: Comparison on carrier rates of ultra-rare LoFs between European and non-European samples in gnomAD exomes and gnomAD genomes** 37

**Supplementary Figure S9: Comparing the high confidence LoF rate in 31,976 unrelated ASD cases with gnomAD exomes and TopMed** 39

**Supplementary Figure S10: Expression signatures of new ASD genes** 41

**Supplementary Figure S11: Calculated cognitive impairment and sex ratio in individuals with ASD in SPARK** 43

**Supplementary Figure S12: Distribution of different types of LoF variants in known ASD genes enriched by de novo variants (DNVs) and comparison with population controls** 44

**Supplementary Figure S13: Empirical relationship between estimated relative risk to ASD and estimated selection coefficient** 46

**Supplementary Figure S14: Burden of de novo and inherited LoFs in genes with high and low LoF mutation rates** 48

**Supplementary Figure S15: Summary of final DNV call sets for SPARK and SSC discovery samples** 51

**Supplementary Figure S16: Evidence of post-zygotic mosaicisms in the final DNV call set** 52

**Supplementary Figure S17: Rare variant workflow and QC strategy** 53

**Supplementary Figure S18: Comparison of inhouse DenovoWEST results on NDD trios with published results** 55

**Supplementary Figure S19: Illustration of pseudo cases and contributing sample sizes in different types of pedigrees** 57

**Supplementary Figure S20: Workflow for variant filtering on rare inherited LoFs** 58

**Supplementary Figure S21: SPARK sample QC: relatedness check and sex validation** 60

**Supplementary Figure S22: SPARK principal component analysis (PCA) and ancestry inference** 62

**Supplementary Figure S23: SPARK self-reported cognitive impairment shows stronger correlation with Vineland score than full-scale IQ** 64

**Supplementary Figure S24: Comparing phenotypes of samples from simplex and multiplex families in SPARK cohort** 65

**Reference** 67

# Supplementary Note

## *Variant-level quality control*

*Data processing*

Saliva was collected using the OGD-500 kit (DNA Genotek) and DNA was extracted at PreventionGenetics (Marshfield, WI). The samples were processed with custom NEB/Kapa reagents, captured with the IDT xGen capture platform, and sequenced on the Illumina NovaSeq 6000 system using S2/S4 flow cells. Samples were sequenced to a minimum standard of >85% of targets covered at 20X. 97% of samples have at least 20x coverage in >95% of region (99% of samples — in 89% of regions). Pending sample availability, any sample with 20X coverage below 88% was re-processed and the sequencing events were merged to achieve sufficient coverage. The Illumina Infinium Global Screening Array v1.0 (654,027 SNPs) was used for genotyping. The average call rate is 98.5%. Less than 1% of samples have a call rate below 90%. Sequencing reads were mapped to human genome reference (hg38) using bwa-mem[1] and stored in CRAM format[2]. Duplicated read pairs in the same sequencing library of each individual were marked up by MarkupDuplicates of Picard Tools[3]. Additional QC metrics for GC bias, insert size distribution, hybridization selection were also calculated from mapped reads by Picard Tools[3]. Mosdepth[4] was used to calculate sequencing depth on exome targets (or 500 bp sliding windows for WGS) and determine callable regions at 10X or 15X coverage. Cross-sample contamination was tested by VerifyBamID[5] using sequencing only mode. Samples were excluded if it has insufficient coverage (less than 80% targeted region with >=20X), shows evidence of cross-sample

contamination (FREEMIX>5%), or discordant sex between normalized X and Y chromosome depth and self/parent reports that cannot be explained by aneuploidy. Variants for each individual were discovered from mapped reads using GATK HaplotypeCaller[6], weCall[7], and DeepVariant[8]. Individual variant calls from GATK and weCall were stored in gVCF format and jointly genotyped across all samples in each sequencing batch using GLnexus[9]. Variants were also jointly discovered and genotyped for individuals of the same family using GATK HaplotypeCaller[6] and freebayes[10], and then read-backed phased using WhatsHap[11]. To verify sample relatedness, identify overlapping samples with other cohorts, and verify sample identity with SNP genotyping data, genotypes of over 110,000 known biallelic SNPs from 1000 Genomes or HapMap projects that have call rate >98% and minor allele frequency (MAF) >1% in the cohort were extracted from joint genotyping VCFs. SNP array genotypes were called by Illumina GenomeStudio. We kept samples with >90% non-missing genotype calls and used genotypes of over 400,000 known SNPs that have call rate >98% and MAF>0.1 for relatedness check and ancestry inference.

*Variant annotations*

The genomic coordinates of QC passed variants were lifted over to hg19 and normalized to the leftmost positions[12]. Functional effects of coding variants were annotated to protein coding transcripts in GENCODE V19 Basic set[13] using variant effect predictor[14]. The gene level effect was taken from the most severe consequences among all transcripts (based on the following priority: LoF>missense>silent>intronic). The pExt (proportion expressed across transcripts) score for each variant is operationally defined as the sum of the expression of all transcripts that include the

variant, normalized by the expression of the gene in all transcripts included in the annotation[15]. We used transcript level expressions in prenatal brain development from Human Developmental Biology Resource[16] to calculate pExt. Missense variants were annotated by pathogenicity scores of REVEL[17], CADD[18], MPC[19] and PrimateAI[20]. Population allele frequencies were queried from gnomAD[21] and ExAC[22] using all population samples. All rare variants were defined by cohort allele frequency <0.001 (or <0.005 for X chromosome variants). To filter for ultra-rare variants, we keep variants with cohort allele frequency <1.5e-4 (or allele count=1) and population allele frequency <5e-5 in both gnomAD[21] and ExAC[22]. LoF variants on each coding transcript were further annotated by LOFTEE[21] (v1.0, default parameters). We also annotated splice site variants by SpliceAI[23], and removed low confidence splice site variants with delta score <0.2 from LoF variants.

*Variant filtering on rare inherited LoFs*

Variant site level QC filters were calibrated using familial transmission information, assuming that false positive calls are more likely to show Mendelian inheritance error (**Supplementary Figure S17A**). Briefly, we first applied a baseline site level filter that favors high sensitivity, then optimized thresholds for filters with additional QC metrics. The selected QC metrics were reviewed first to determine a small number of optional thresholds. Then the final set of QC parameters were optimized from a grid search over the combinations of available thresholds such that: 1. presumed neutral variants identified from parents (silent variants or variants in non-constrained genes) shows equal transmission and non-transmission to offspring; 2. rates of neutral variants are similar in different sample groups from the same population ancestry; 3. vast majority

variants identified in trio offspring are inherited from parents. In case when multiple sets of QC thresholds give similar results, priority will be given to the set that also recovers maximum number of DNV calls in trio offspring. The optimized filtering parameters were used in final QC filters to generate analysis-ready variants.

For a rare coding variant initially annotated as LoF (including stop gained, frameshift, or splice site), we searched for nearby variants on the same haplotype (within 2bp for SNVs or 50bp for indels). If nearby variants can be found, they were merged to form MNVs or complex indel and re-annotated to get the joint functional effect. If the joint effect was not LoF, then the original variant was removed from LoF analysis.

Standing LoFs are notoriously fraught with variant calling artefacts, low confidence LoFs that escape nonsense mediated decay or do not affect splicing[24-26], or LoFs that only affect transcripts that have low expression in disease relevant tissues[27]. In constrained genes, we found about 6% QC passed variant calls initially annotated as LoFs are part of non-LoF MNV or frame-restoring indels, in contrast to <1% in dnLoFs (**Supplementary Figure S20A**). To prioritize high confidence standing LoFs, we applied of LOFTEE/pExt and allele frequency filters. Using pExt>=0.1 in developing brain removes more than 1/3 of LoFs without changing the over-transmission rate to affected offspring. Further applying ultra-rare allele frequency filter (allele frequency<1.5e-4 or singleton in cohort and <5e-5 in populations) removes additional 11% standing LoFs with minimal changes to over-transmission rate. Together, close to half of standing LoF variants are removed that does not contribute to ASD in offspring. Although further increasing pExt threshold to 0.9 will reduce over-transmission rate, it is likely that optimal pExt threshold is gene-specific and we may underestimate fraction of standing

LoFs that does not contribute to ASD. In comparison, the same set of filters only removes 3% dnLoFs and 5% dnDmis in ASD, 3% dnLoFs and 2% dnDmis in other NDD with minimal changes to rate difference between affected and control trios (**Supplementary Figure S20B**, **Figure 2A**). LoFs in pseudo cases is a mixture of de novo and inherited LoFs, and as expected 25% of them are removed by the same filters which do not change rate difference between cases and pseudo controls (**Figure 2B**). We used ultra-rare high confidence (pExt>=0.1) standing LoFs in transmission analysis.

Among QC passed rare variant calls that were initially annotated as LoFs in 5,754 constrained genes (ExAC pLI>=0.5 or top 20% LOEUF), 6% of standing LoFs in 20,491 unaffected parents are part of non-LoF multi-nucleotide variations (MNVs) or frame-restoring indels. In contrast, we observed less than 1% such variants in *de novo* calls.

pExt for LoF variants was calculated by the proportion of expression level of transcripts that harbor HC LoFs evaluated by LOFTEE over all transcripts included in the analysis. Thus, the pExt filter for LoFs already incorporated LOFTEE annotations. The baseline filter to analyze rare, inherited LoFs and LoFs of unknown inheritance is pExt>=0.1. To refine gene-specific pExt threshold in the second stage, we selected 95 known ASD/NDD genes plus a newly significant DNV enriched gene *MARK2* which harbor at least four *de novo* LoF variants in combined ASD and other NDD trios, and for each gene choose the pExt threshold from {0.1,0.5,0.9} that can retain all *de novo* LoF variant with pExt>=0.1 (**Supplementary Table S17**).

## *Sample-level quality control*

*Relatedness and Ancestry*

We used KING[28] to calculate statistics for pairwise sample relatedness from genotypes of known biallelic SNPs, and validated participant-reported familial relationships (**Supplementary Figure S21A-B**). The relatedness analysis also identified cryptically related families that are connected by unreported parent-offspring or full sibling pairs. Pedigrees were reconstructed manually from inferred pairwise relationships and validated by PRIMUS[29] and we used inferred pedigree for all analyses. Sample sex was validated by normalized sequencing depths or array signal intensities of X and Y chromosomes which also identified X and Y chromosome aneuploidies (**Supplementary Figure S21C-D**). To infer genetic ancestry, we first performed principal component (PC) analysis on SNP genotypes of non-admixed reference population samples from 1000 Genomes Projects[30] (Africans, Europeans, East Asians and South Asians) and Human Genome Diversity Project[31,32] (Native Americans), then projected SPARK samples onto PC axes defined by the five reference populations using EIGNSOFT[33] (**Supplementary Figure S22**). The projected coordinates on first four PC axes were transformed into probabilities of five population ancestries using the method of SNPweights[34]. The inferred ancestral probabilities show general concordance with self-reported ethnicities (**Supplementary Figure S22B**). Samples were predicted from a reference population if the predicted probability was >=0.85.

*Phenotype*

The phenotypes of participants are based on self- or parent-report provided at enrollment and in a series of questionnaires from the Simons Foundation Autism

Research Initiative database, SFARI Base. We used SFARI Base Version 4 for the discovery cohort and Version 5 for the replication cohort. In the discovery cohort, information about self-reported cognitive impairment (or ID/developmental delay) was available for 99.2% of ASD cases and 83.5% of other family members at recruitment or from the Basic Medical Screening Questionnaire available on SFARIbase. For phenotype-genotype analyses in individuals with variants in specific ASD risk genes, we defined an individual as having cognitive impairment if 1) there was self- or parent-report of cognitive impairment at registration or in the Basic Medical Screening Questionnaire, 2) the participant was at or over the age of 6 at registration and was reported to speak with less than full sentences or the participant was at or above age 4 at registration and reported as non-verbal at that time, 3) the parent reported that cognitive abilities were significantly below age level, 4) the reported IQ or the estimated cognitive age ratio (ratio IQ[35,36]) was <80 or 5) the parent reported unresolved regression in early childhood without language returning and the participant does not speak in full sentences. The continuous full-scale IQ was imputed based on a subset of 521 samples with full scale IQ and phenotypic features by the elastic net machine learning model[37]. In a subset of cases for which full-scale IQ data or standardized Vineland adaptive behavior scores (version 3) was available, we found self-reported cognitive impairment shows higher correlation with Vineland score than full-scale IQ (**Supplementary Figure S23**). ASD cases with self-reported cognitive impairment were defined as Cognitively Impaired cases, and other cases as Not Cognitively Impaired cases. Other non-ASD family members were considered as unaffected if they were also not indicated to have cognitive impairment. In total of 18.5% families, proband has at

least one first-degree relative with ASD who was recruited in the study and/or reported by a family member. Those families were referred to as multiplex, and other families with only a single ASD individual as simplex. The majority (>85%) of affected relative pairs in multiplex families were siblings. Multiplex families have slightly lower male-to-female ratio and lower proportion of cognitive impairment among affected offspring (**Supplementary Figure S24A-B**). In comparison, only 1% of parents in the discovery cohort are affected of which two thirds are females and less than 3% have cognitive impairment (**Supplementary Figure S24A-B**). In addition, non-ASD family members in multiplex families show significantly higher frequency of self-reported cognitive impairment, learning/language disorders, other neuropsychiatric conditions, and other types of structural congenital anomalies (**Supplementary Figure S24C**). Non-ASD parents in multiplex families also have lower educational attainment (**Supplementary Figure S24D**).

### *Copy number variants*

Copy number variants (CNVs) were called from exome read depth using CLAMMS[38]. CNV calling windows used by CLAMMS were created from exome targets after splitting large exons into equally sized windows of roughly 500bp. Calling windows were annotated by average mappability score[39] (100mer) and GC content assuming average insert size of 200. Depths of coverage for each individual on the windows were calculated using Mosdepth[4] and then normalized to control for GC-bias and sample's overall average depth. Only windows with GC content between 0.3 and 0.75 and mappability >=0.75 were included in further analyses. For each given sample, we used two approaches to reduce the dimension of sample's coverage profile and automatically

selected 100 nearest neighbors of the sample under analysis as reference samples. The first approach used seven QC metrics calculated by Picard Tools from aligned reads as recommended by the CLAMMS developer[38], we further normalized those metrics in the cohort by its median absolute deviation in the cohort. The second approach used singular value decomposition of the sample by read-depth matrix to compute the coordinates of the first 10 principal components for each sample.
Model fitting and CNV calling for each individual using custom reference samples were performed using default parameters. From raw CNV calls, neighboring over-segmented CNVs of the same type were joined if joined CNVs include over 80% of the calling windows of original calls. For each sample, we kept CNV calls made from one set of reference samples that have smaller number of raw CNV calls. Outliers with excessive raw CNV calls (>400) were removed. For each CNV, we counted the number of CNVs of the same type in parents that overlap >50% of the calling windows. High-quality rare CNVs were defined as <1% carrier frequency among parents and have Phred-scaled quality of CNV in the interval >90. We queried high-quality rare copy number deletions to look for additional evidence to support new genes.

## *Other ASD cohorts*

*Simon Simplex Collection (SSC)*

SSC collected over 2,500 families with only one clinically confirmed ASD cases who have no other affected first or second degree relatives as an effort to identity *de novo* genetic risk variants for ASD[40]. SSC data have been published before[41-44]. Here we included 10,032 individuals including 2,633 cases with exome or WGS data available and passed QC. The data were reprocessed using the same pipeline as SPARK. For 91

trios that are not available or incomplete, we collected coding DNVs from published studies[41,43]. In analysis to associate genetic variants with phenotype severity, we used standardized Vineland adaptive behavior score to group affected cases because it shows higher correlation than full-scale IQ with self-reported cognitive impairment in SPARK (**Supplementary Figure S23, Supplementary Figure S24**). Cases with cognitive impairment in SSC were defined by Vineland score<=70, and cases with no cognitive impairment by score>70.

*Autism Sequencing Consortium (ASC)*

ASC is an international genomics consortium to integrate heterogenous ASD cohorts and sequencing data from over 30 different studies[45]. Individual level genetic data are not available. We included 4,433 published trios (4,082 affected and 351 unaffected) merged from two previous studies[46,47] for DNV analysis. To define low and high functioning cases, we used binary indicator of ID which was available for 66% of cases. Families with multiple affected trios are considered multiplex, others are simplex.

*MSSNG*

The MSSNG initiative aims to generate WGS data and detailed phenotypic information of individuals with ASD and their families[48]. It comprehensively samples families with different genetic characteristics in order to delineate the full spectrum of risk factors. We included 3,689 trios in DB6 release with whole genome DNV calls are available and passed QC in DNV analysis, of which 1,754 trios were published in the previous study[48]. A total of 3,404 offspring with a confirmed clinical diagnosis of ASD were included as cases. Among individuals without a confirmed ASD diagnosis, 222 who did not show broader or atypical autistic phenotype or other developmental disorders were used as

part of controls. Multiplex families were defined as families having multiple affected siblings in sequenced trios or in phenotype database. Information about cognitive impairment was not available at the time of analysis.

## Supplementary Table Legends

Table S1. Cohorts and number of trios included in *de novo* variants analysis.

Table S2. Full list of 618 known dominant or X-linked ASD or NDD genes.

Table S3. Genes with p-value < 0.001 in DeNovoWEST analysis of de novo variants in the Stage 1 data. The table summarizes numbers of different types of de novo variants in each gene and results from DenovoWEST test in 16,877 ASD trios.

Table S4. Number of unaffected parents and offspring in trios and duos used in transmission disequilibrium analysis.

Table S5. Gene sets memberships. Gene set enrichment analyses for de novo and rare, inherited LoFs used 5,754 constrained genes (gnomAD LOEUF top 20% or ExACpLI >=0.5) as background. Their memberships in 25 gene sets of 5 categories are listed in this table.

Table S6. Transmission disequilibrium test (TDT) of rare, inherited LoFs in the discovery cohort. For each autosomal gene, rare, inherited LoFs that passed different allele frequency and pExt filters were identified 20,491 unaffected parents and evaluated over-transmission to ASD offspring in 9,504 trios and 2,966 duos. And for each non-PAR chrX gene, LoFs were identified in 11,354 unaffected mothers and evaluated over-transmission to affected sons in 9,883 duos. A total of 260 genes were prioritized for replication (15 overlap with top de novo enriched genes).

Table S7. DeNovoWEST analysis of de novo variants among 404 selected genes in the Stage 1 & 2 data. A total of 404 genes were selected for the combined analysis,

including 159 top de novo enriched genes (A) or additional 245 genes prioritized from transmission disequilibrium test of rare, inherited LoFs (B). Enrichment of de novo variants in those genes were tested in trios from combined discovery and replication cohort.

Table S8. Effective number of cases and controls in case-control comparison.

Table S9. Meta-analysis of selected autosomal genes in combined stage 1 and 2 cohort. Gene-based meta-analysis was performed by combining p-values from enrichment of all de novo variants, transmission disequilibrium test (TDT) of rare, inherited LoFs from unaffected parents to affected offspring, and increased rate of LoFs in cases vs population controls. A total of 391 autosomal genes selected for replication were included in meta-analysis. Only high confidence (HC) LoFs were included in TDT and cases vs population controls comparisons. We used gene-specific pExt thresholds and removed curated non-LoFs if any to prioritize HC LoFs; for other genes, we used default pExt>=0.1 filter. LoF rates in population controls were tallied from summary level data of gnomAD exomes (v2.1.1) and TopMed (Freeze 8). For controls from gnomAD, we used samples that are not ascertained for neurological or psychiatric phenotypes (non-neuro subset) to generate final results. The following allele frequency filter was used: ultra-rare variants have cohort allele frequency<1.5e-4 and population allele frequency<5e-5.

Table S10: Ancestry specific case-control analysis for five novel genes

Table S11. Estimated average relative risk of rare LoFs of selected autosomal genes.

Table S12. Phenotypic information on individuals with HC LoFs in novel, exome-wide significant ASD risk genes and individuals with HC LOFs in 5 well-established ASD risk genes (1 means yes, condition is present. 0 means no, condition is not present. NA means the information is not known).

Table S13. Gene-wise scores for each mechanistic archetype.

Table S14. Enrichments of STRING clusters for each archetype. Each STRING cluster (a binary label across 1,776 genes in our embedding space) was predicted as a function of the six archetype scores we derived.  The significance of each model parameter was assessed and the corresponding p-value is reported if the coefficient was positive (enrichment) or set to 1 if the coefficient was negative.

Table S15. Enrichments of MSigDB gene sets for each archetype. Colors indicate different archetype.

Table S16. Details about software tools and their parameter settings used in data processing.

Table S17. Gene-specific pExt thresholds for 96 de novo LoF (dnLoF) enriched genes. We selected 96 known or DenovoWEST exome-wide significant genes that are in the top 30% of gnomAD LOEUF scores and have more than 4 dnLoFs in 23,053 ASD trios. For each gene, a gene-specific pExt threshold was selected from {0.1, 0.5, 0.9} such that all dnLoFs with pExt>=0.1 were be retained. For selected genes that were manually reviewed, we further removed curated non-LoF variants in gnomAD.  (A) Compared with the baseline filter of pExt>=0.1, applying gene-specific pExt thresholds and removing curated non-LoFs further filtered out 19% of rare, inherited LoFs in selected genes that

have minimal contribution to transmission disequilibrium to affected offspring in 15,603 trios and 4,925 duos. (B) Those filters also further removed an additional 3~4% LoFs in cases after the baseline filter pExt>=0.1, while retaining similar LoF rate differences compared to pseudo-controls. (C) Gene specific pExt thresholds for 96 selected genes.

Table S18. Burden of de novo variants (DNVs). Burden of DNVs were evaluated by comparing observed with expected rates calculated from baseline mutation rates. The table summarizes burdens of different types of DNVs in constrained (pLI>=0.5) and non-constrained genes (pLI<0.5) and the corresponding positive predictive values that are used as weights in DeNovoWEST.

Table S19. Summary statistics and statistical test results in figures

## Supplementary Data Legends

Supplementary Data 1: Annotated de novo coding variants in ASD discovery cohorts

Supplementary Data 2: Annotated de novo coding variants identified in SPARK replication cohort

Supplementary Data 3: Annotated de novo coding variants collected from other NDD studies

Column Description:

Cohort: Cohort of the sample

IID: Sample ID

Sex: Sex assigned at birth

Pheno: ASD affection status

DNASource: Source of DNA

VarID: Variant ID in format "Chrom:Position:Ref:Alt" (hg19)

Chrom,Position,Ref,Alt: Genomic coordinate (in hg19) of the variant and reference/alternative alleles

Context: Tri-nucleotide sequence context(SNV only)

GeneID: Ensembl gene ID from GENCODE V19 Basic Set (Ensembl release 75). When a variant is mapped to multiple overlapping genes, gene IDs are semi-colon separated, and all other gene level annotations will appear in the same order of gene IDs and separated by semi-colon.

HGNC: Gene symbol based on HGNC 2018-07-22

ExACpLI, LOEUFbin, Arisk: Gene level metrics: ExAC pLI, gnomAD LOEUF decile, and A-risk prediction score

GeneEff: The gene level effect, defined as the most severe consequence among all protein: coding transcripts. Annotations to multiple overlapping genes are semi-column separated.

TransCount: Number of transcripts that are annotated. All protein coding transcripts including up and down-stream 5000 bp regions that overlap with the variant are included in annotation.

TransIDs: Ensembl IDs of annotated transcripts (from GENCODE V19 Basic Set). Different transcripts for each gene are comma separated, all other transcript level annotations will appear in the same order of transcript IDs and separated by comma. Transcripts from different genes appear in the same order as gene IDs and are separated by semi-column.

TransEffs, cDNAChg, CodonChg, AAChg: Transcript level annotations: functional consequences, cDNA, codon and amino acid changes

REVEL, MPC, PrimateAI: Missense pathogenicity prediction scores: REVEL, MPC, and PrimateAI

CADD13: PHRED-scaled CADD score v1.3 and truncated that only show values >= 20

ExAC_ALL, gnomADexome_ALLPopulation allele frequencies from all samples in ExAC and gnomAD exomes. All variants regardless their filtering flags were used to query allele frequencies.

LoF,LoF_filter,LoF_flags: Loftee (v1) annotations using default parameters. This annotation is transcript specific. LoF is the final classification (HC or LC) for putative LoF variants in each transcript. For variants that are classified as LC, failed filters for

each transcript will be listed in LoF_filter. Additional flags will be listed in LoF_flags. See loftee doc for details.

pExt_GTExBrain, pExt_HBDR: pExt metrics. It is operationally defined as the sum of expression levels of transcripts that have the same functional consequences as GeneEff divided by the transcription levels of all transcripts used in the annotation. For LoF variants, only transcripts affected by HC LoF (by loftee) are included in pExt calculation. Two sets of expression data are used: GTEx v6 brain subset and Human Developmental Biology Resource (HBDR).

HGNCv24, DS_AG, DS_AL, DS_DG, DS_DL, DP_AG, DP_AL, DP_DG, DP_DL: SpliceAI annotations: gene predicted to be influence by the variant (symbols from GENCODE V24) and predicted distances and probabilities of splice site gain or loss events. See SpliceAI doc for details.

# Supplementary Figure S1: Transmission disequilibrium of LoF variants of chromosome X genes

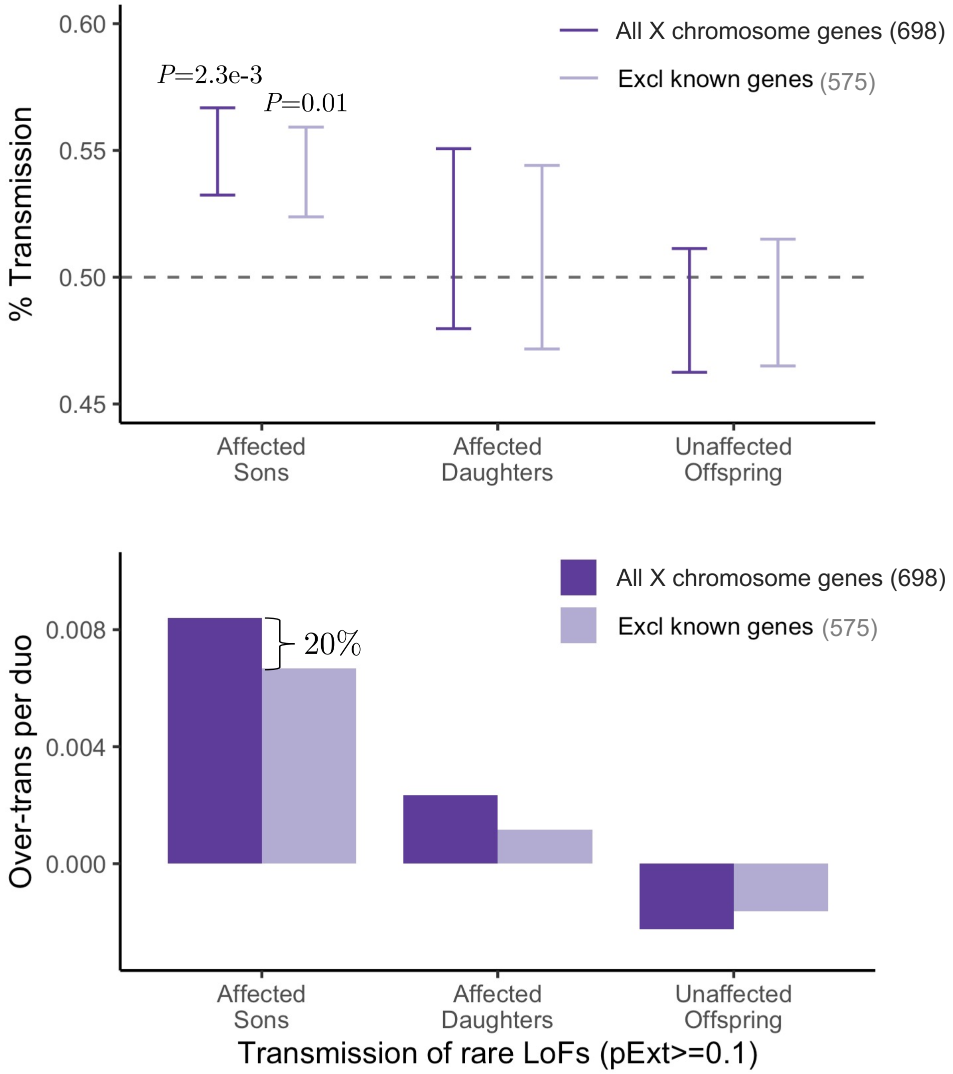


The burden of inherited LoFs on non-PAR part of chrX was evaluated by analyzing the transmission disequilibrium of rare (cohort AF<0.001 and population AF<2e-4) rare, inherited LoFs (pExt>=0.1) identified unaffected mothers. Data are presented as mean values +/- standard errors as error bars. Across all chrX genes, only the proportion of

transmission to affected son are significantly above 50% and remain so after excluding known ASD/NDD genes. We observed no significant over-transmission of chrX LoFs to affected daughter or unaffected offspring.

# Supplementary Figure S2: Transmission disequilibrium of deleterious missense (D-mis) variants

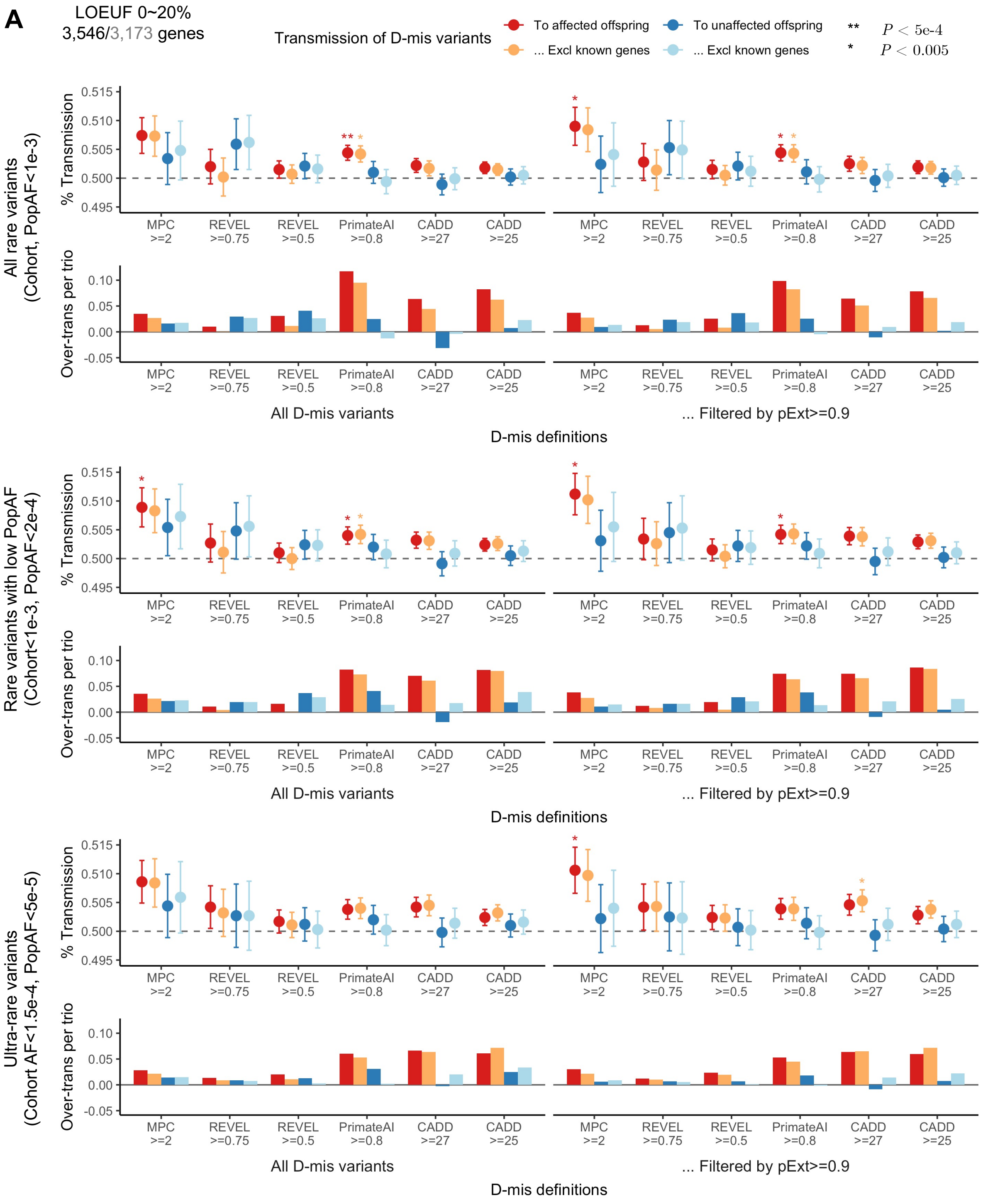

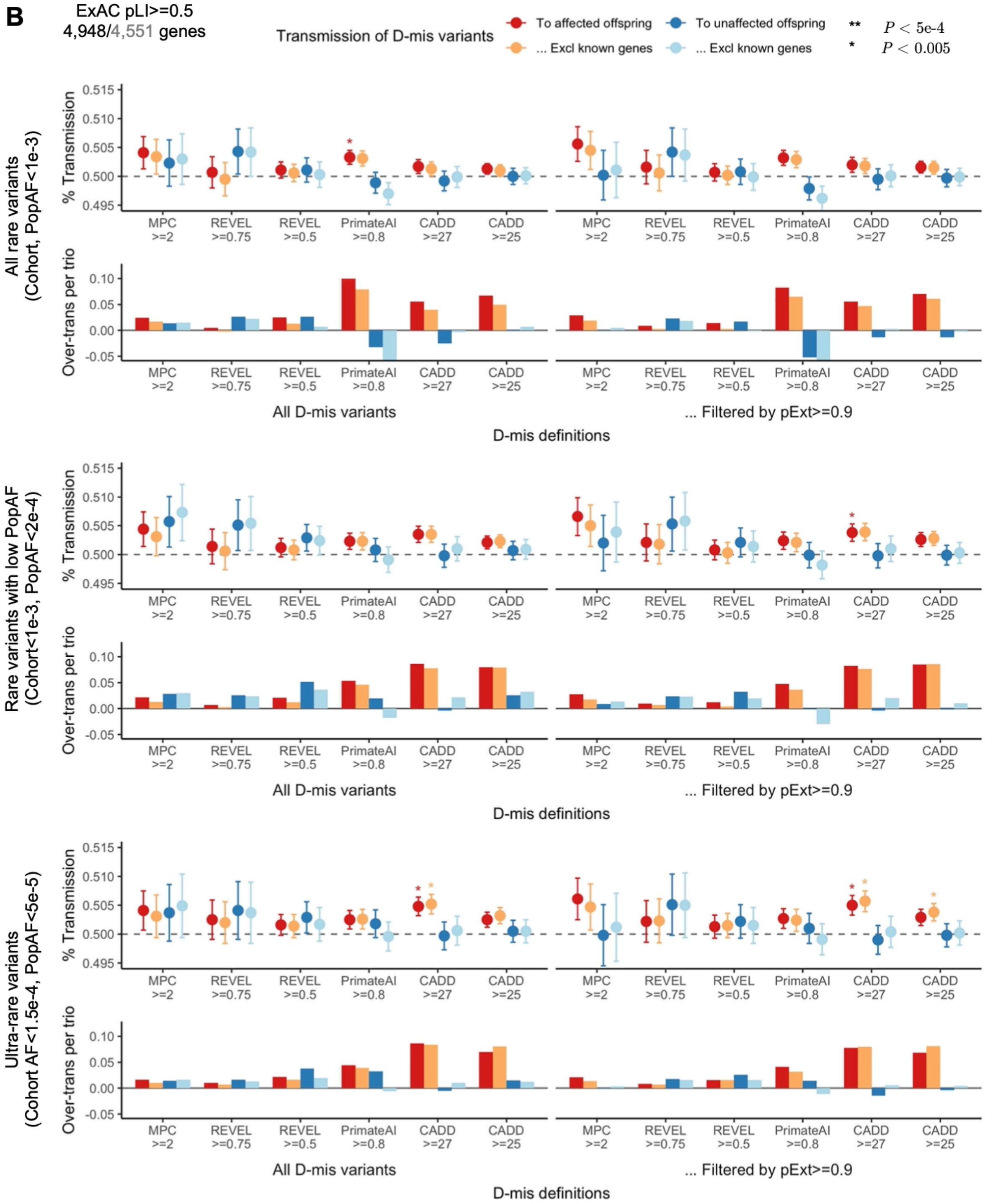


The transmission disequilibrium signals of rare, inherited D-mis variants are generally weaker than LoFs and sensitive to the definition of D-mis and the choice of gene set. For example, in genes of top 20% gnomAD LOEUF (n=3,526; A), Significant over-

transmission to affected offspring was observed for rare, inherited D-mis variants defined by MPC>=2, especially those that are further filtered by pExt>=0.9 (P<0.005). PrimateAI>=0.8 prioritized more than two times D-mis variants than MPC>=2 and show significant over-transmission to affected but with lower magnitude than MPC>=2. As a comparison in constrained genes with ExAC pLI>=0.5 (n=4,948; B), over-transmission of D-mis variants defined by MPC>=2 become non-significant. The ultra-rare inherited D-mis defined by CADD>=27 shows strong evidence of over-transmission. Most significant transmission disequilibrium signals remain significant after removing known ASD/NDD genes. Data are presented as mean values +/- standard errors as error bars in (A) and (B).

# Supplementary Figure S3: Number of genes in each gene set and pairwise overlaps between gene sets before (A) and after (B) excluding known ASD/NDD genes

A

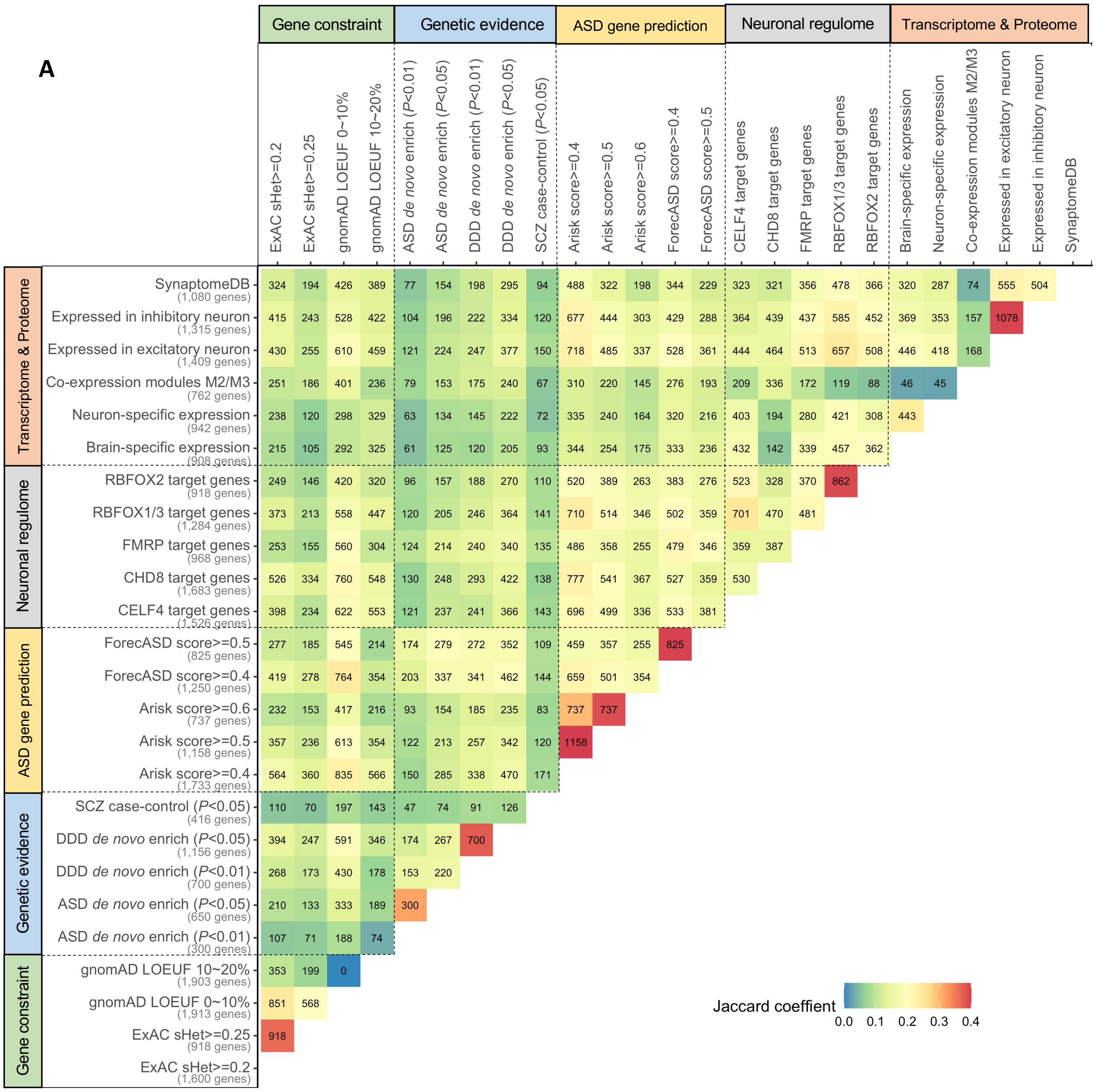

**B**

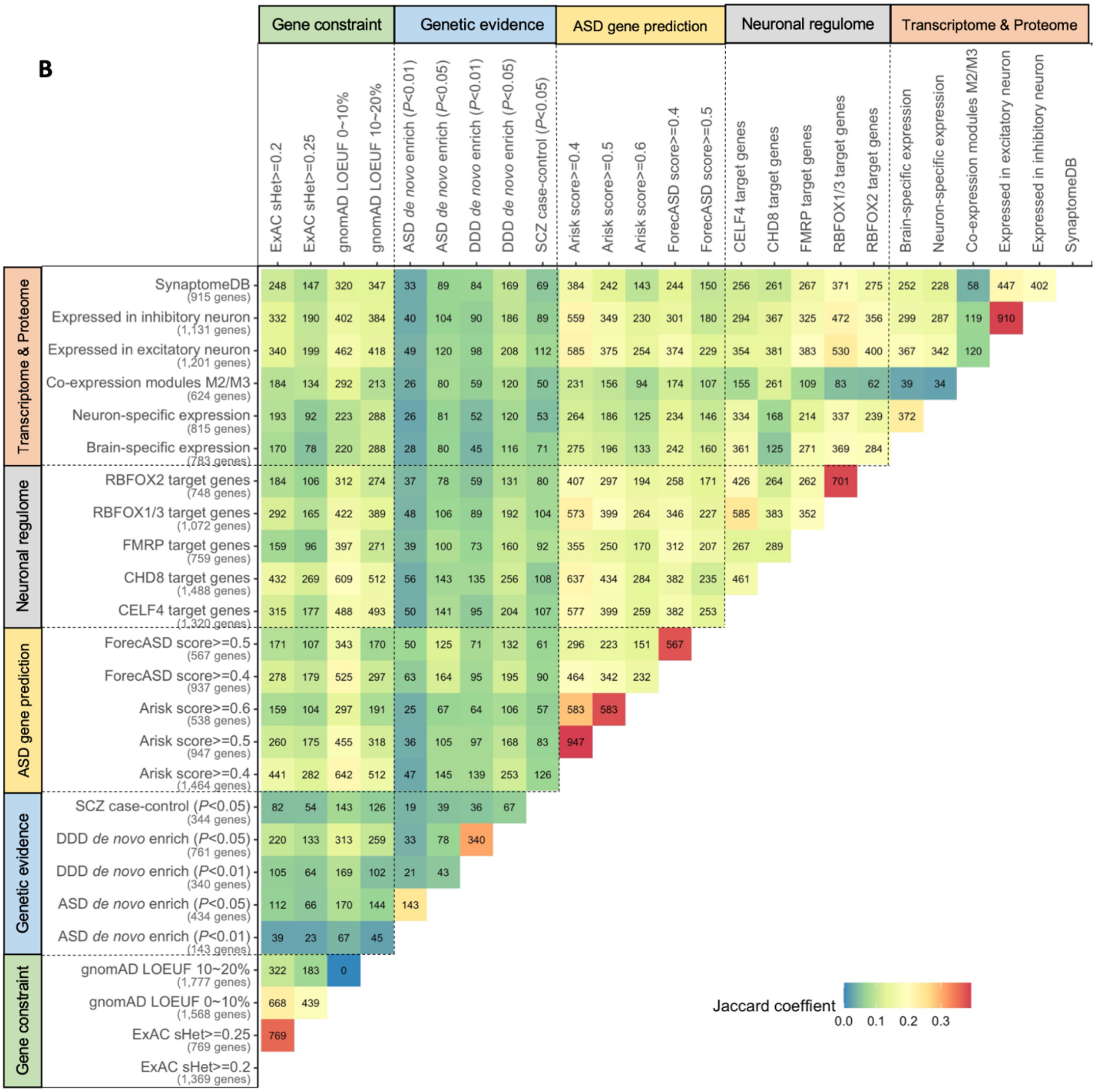


Similarities between gene sets are measured and visualized by Jaccard coefficients.

## Supplementary Figure S4: Inherited LoF variants in genes prioritized by A-risk are not associated with phenotype severity in cases

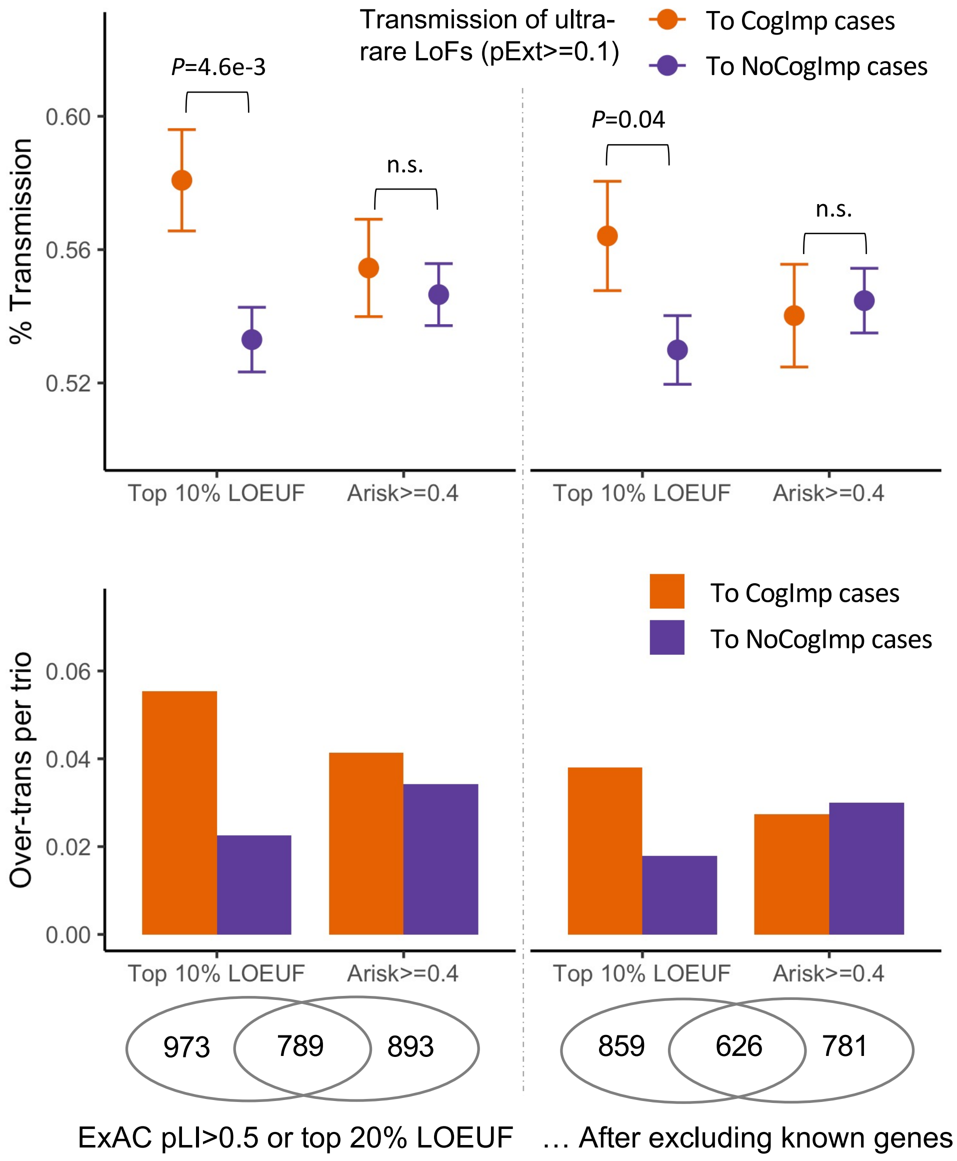


Transmission disequilibrium signals are significantly enriched in genes at top 10% gnomAD LOEUF metrics (n=1,762) or having A-risk score>=0.4 (n=1,682; Extended

data fig. 2). Number of autosomal genes in each set are shown as Venn diagram below the plot. Despite of over 700 overlapping genes (over 600 after removing known genes) in the sets, ultra-rare LoFs with pExt>=0.1 in genes at top 10% gnomAD LOEUF shows significantly higher proportion of transmission to with cognitive impairment ASD cases, whereas those in genes with A-risk>=0.4 show similar proportion of transmission to cases with or without cognitive impairment. Data are presented as mean values +/- standard errors as error bars.

# Supplementary Figure S5: Comparing carrier rates of LoFs between pseudo-controls and three panels of population-based references for genes selected for replication

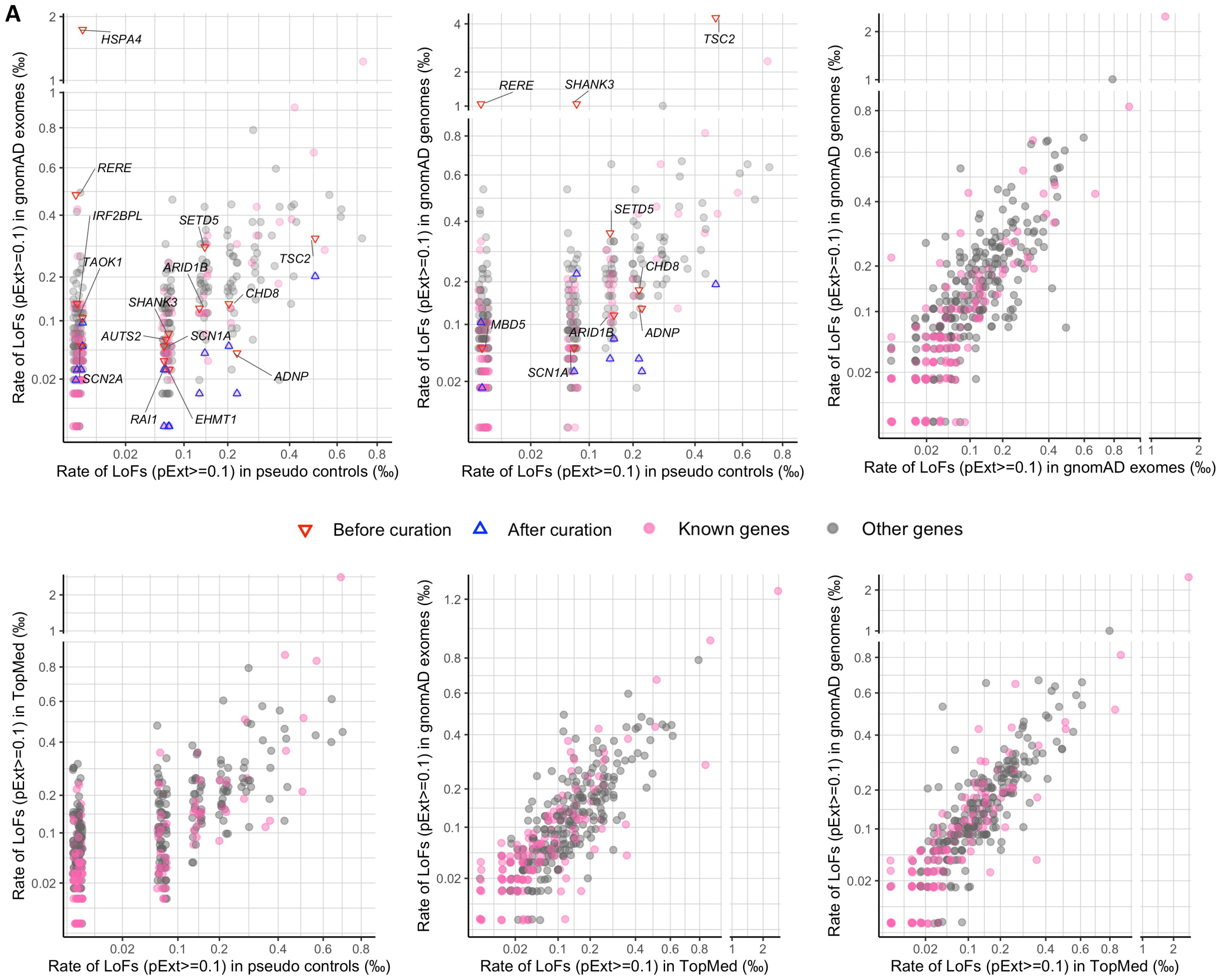

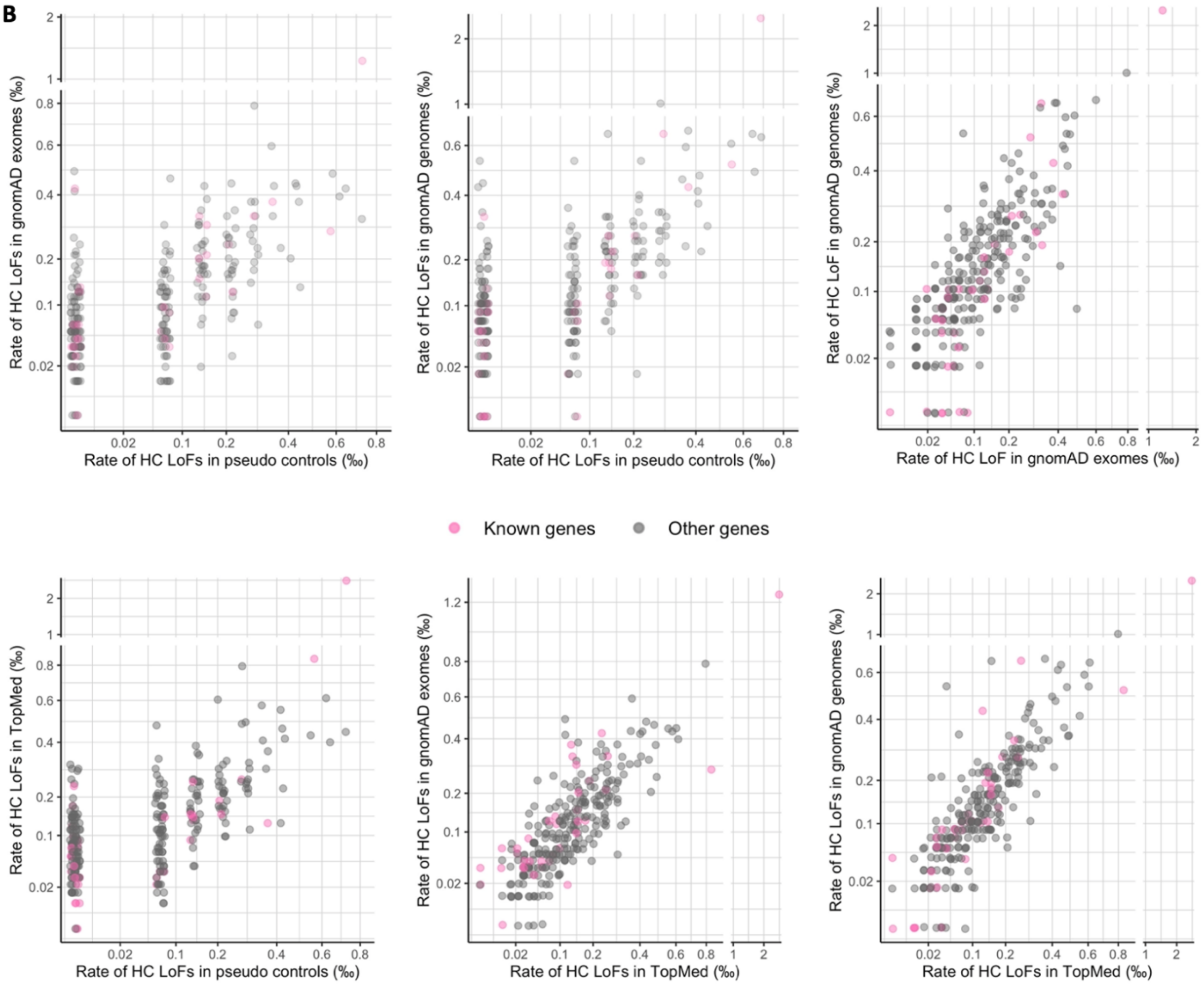


Prioritized gene in top 30% gnomAD LOEUF were used in meta and mega analysis; shown in this plot are 367 autosomal genes. Carrier rates are estimated from 14,128 unrelated pseudo-controls, 104,068 gnomAD exome samples (non-neuro subset), 67,442 gnomAD genome samples (non-neuro subset), and 132,345 TopMed samples. (A) LoFs were filtered by pExt>=0.1. Selected genes whose carrier frequencies change by over 1/3 in gnomAD after manual curation are highlighted. (B) LoFs in de novo LoF enriched genes were further filtered by gene-specific pExt thresholds.

**Supplementary Figure S6: Empirical relationship between haploid LoF mutation rate, cumulative allele frequency (CAF) of HC LoFs, and fraction of de novo LoFs in ASD cases**

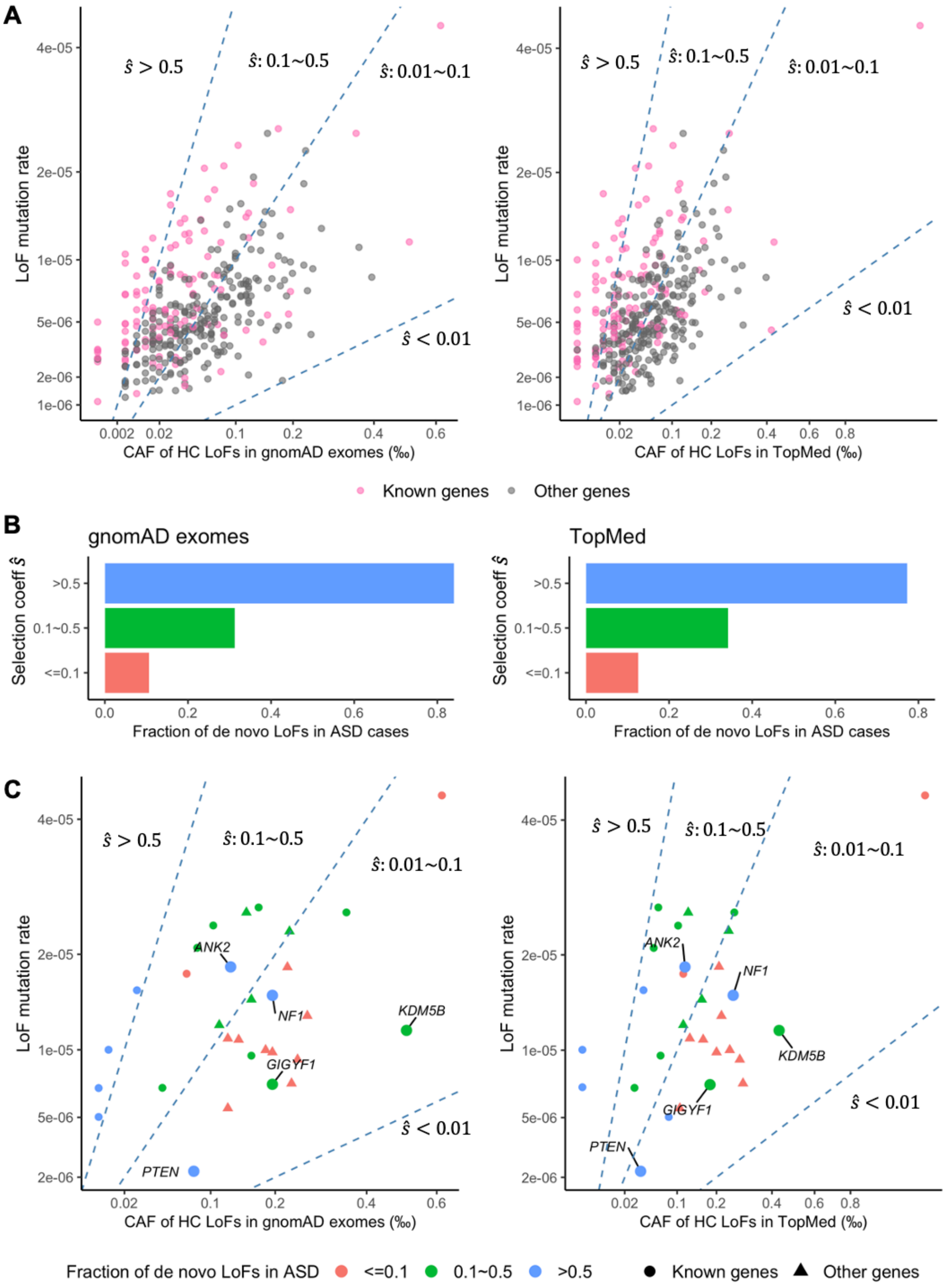

Selection coefficient can be estimated as the ratio of mutation rate over CAF ($\hat{s} = \mu/\hat{f}$). (A) Comparing haploid LoF mutation rate with CAFs of HC LoFs in populations on 367 autosomal genes selected for replication and among top 30% gnomAD LOEUF. CAFs in populations are estimated from gnomAD exomes (125,748 individuals), gnomAD genomes (76,156 individuals), and TopMed (132,345 individuals). Three dashed lines in each plot demarcate the quadrant into areas different estimated selection coefficient ($\hat{s}$: <0.01, 0.01~0.1, 0.1~0.5, and >0.5). Almost all genes have $\hat{s}$>0.01 and known ASD/NDD genes tend to have higher $\hat{s}$. (B) We grouped genes into three bins of $\hat{s}$ (0.01~0.1, 0.1~0.5, and >0.5) and tallied the number of de novo and inherited LoFs in 32,024 unrelated ASD cases. Genes in higher $\hat{s}$ bin have higher proportion of LoFs that are de novo in cases. (C) The same as (A) but only show 30 genes with more than 10 LoFs with inheritance information in unrelated ADS cases. Gene are color coded by the observed proportion of de novo LoFs. There is a general concordance between $\hat{s}$ and fraction of de novo LoFs in these genes. Five genes that also harbor de novo LoFs in control trios (Supplementary Tab 4) are highlighted. These genes have likely under-estimated LoF mutation rates and shows higher proportion of de novo LoFs than $\hat{s}$ estimated from presumed mutation rates.

## Supplementary Figure S7: In genes selected for replication, most LoFs are ultra-rare

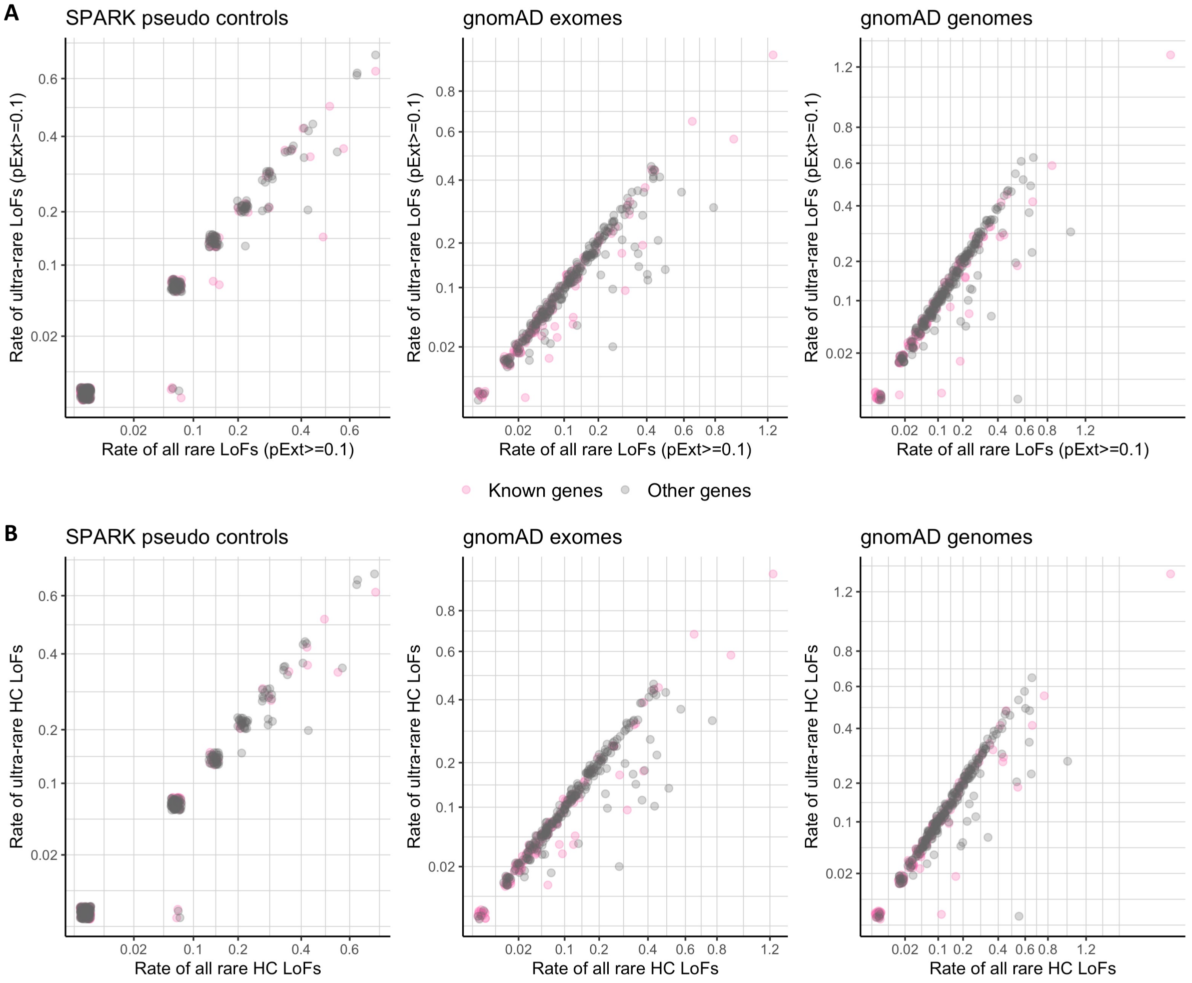


Prioritized gene in top 30% gnomAD LOEUF were used in meta and mega analysis, of which 367 are on autosomes and shown in the plot. We compared carrier rates of all LoFs versus ultra-rare LoFs observed in 14,128 SPARK pseudo controls, 104,068 gnomAD exomes (non-neuro subset), and 67,442 gnomAD genomes (non-neuro subset). Ultra-rare variants are defined by cohort allele frequency <1.5e-4 (or singleton in the cohort) and population allele frequency <5e-5. Most genes are shown along or

close the diagonal line, suggesting that most LoFs in those genes are ultra-rare and originated from recent mutational events. (A) LoFs were filtered by pExt>=0.1. (B) LoFs in de novo LoF enriched genes were further filtered by gene-specific pExt thresholds.

## Supplementary Figure S8: Comparison on carrier rates of ultra-rare LoFs between European and non-European samples in gnomAD exomes and gnomAD genomes

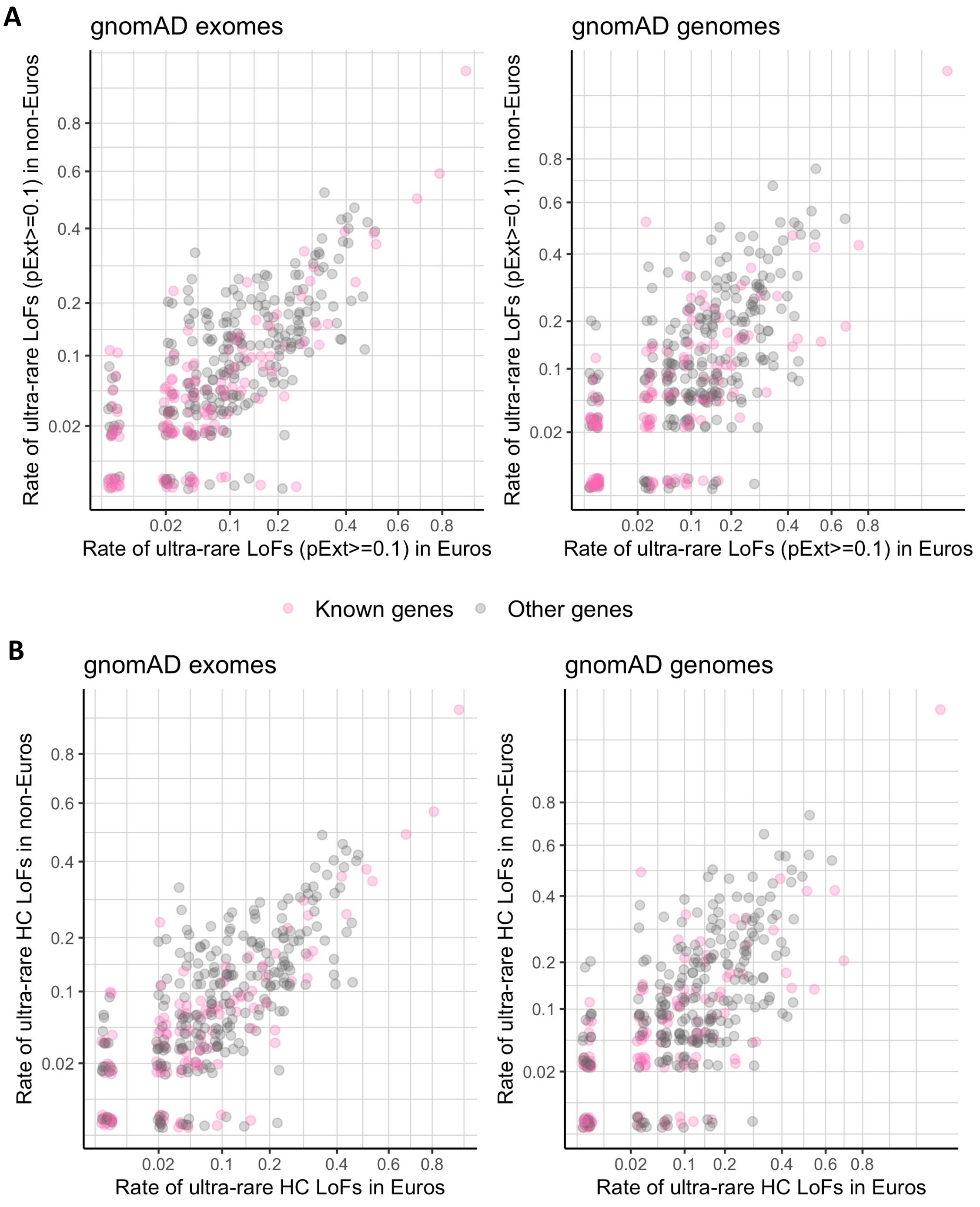

For the same 367 genes shown in Supplementary Figure S9, we compared carrier rates of ultra-rare LoFs between European and non-European samples in gnomAD exomes (44,779 Europeans, 59,289 non-Europeans) and gnomAD genomes (31,966 Europeans, 35,476 non-Europeans). Carrier rates of ultra-rare LoFs in European and non-European population samples are higher correlated, consistent with their recent mutational origin and insensitive to population demographic history. (A) LoFs were filtered by pExt>=0.1. (B) LoFs in de novo LoF enriched genes were further filtered by gene-specific pExt thresholds

# Supplementary Figure S9: Comparing the high confidence LoF rate in 31,976 unrelated ASD cases with gnomAD exomes and TopMed

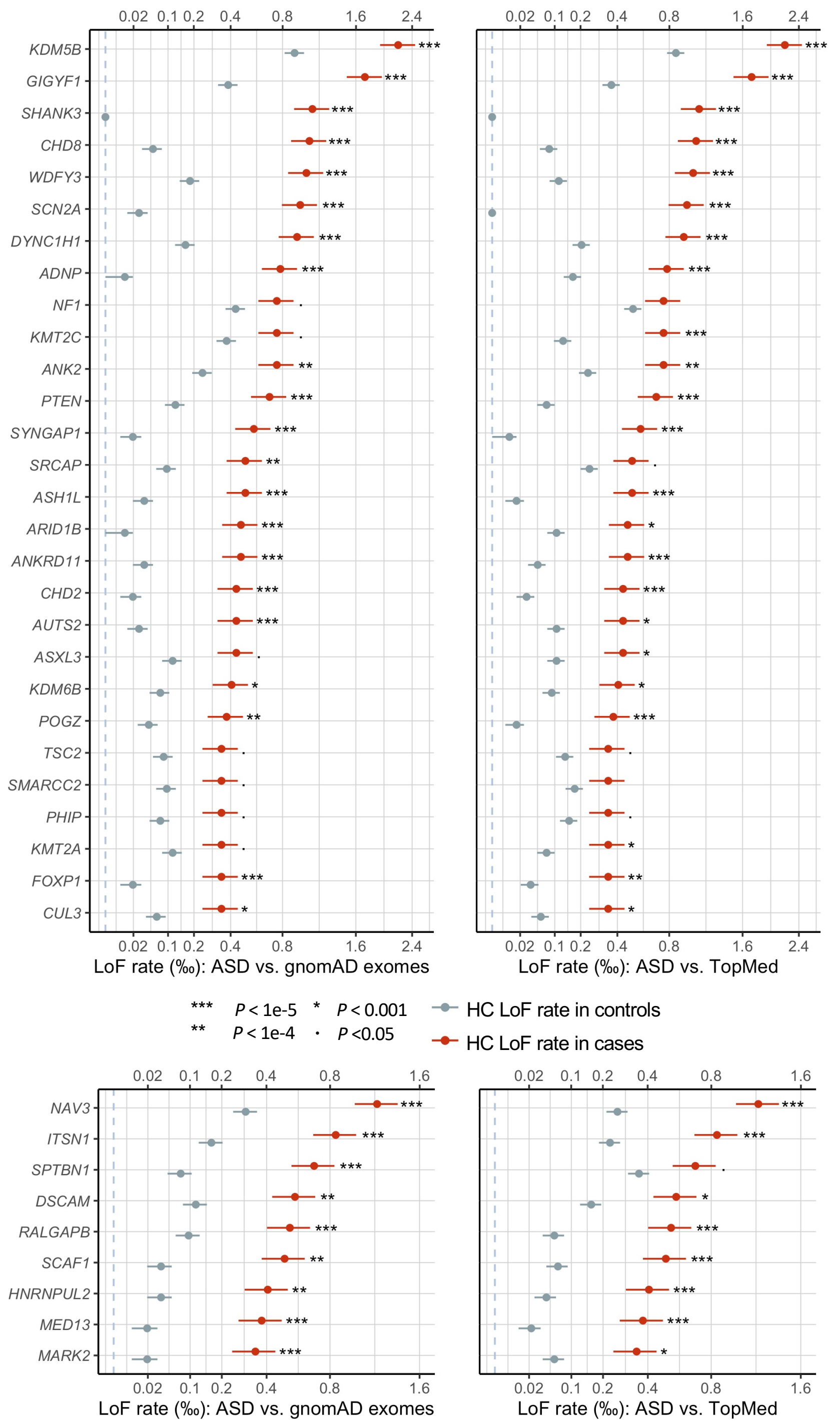

Horizontal bars are presented as mean values +/-. The upper panel shows 28 known ASD/NDD genes in which LOEUF scores are in the top 30% of gnomAD, have a p-value for enrichment among all DNVs (p <9e-6) in 23,039 ASD trios, and have more than 10 LoFs. The lower panel shows 9 additional ASD risk genes that achieved a p-value of <9e-6 in Stage 2 of this analysis. The majority of genes in the lower panels harbor more inherited LoFs than de novo variants. All five novel genes (Table 1) are shown in the lower panel. Note that the x-axes of LoF rates are in the squared root scale. Poisson test was used to compute the p values for comparing the LoF rate between unrelated ASD cases and gnomAD exomes or TopMed controls.

# Supplementary Figure S10: Expression signatures of new ASD genes

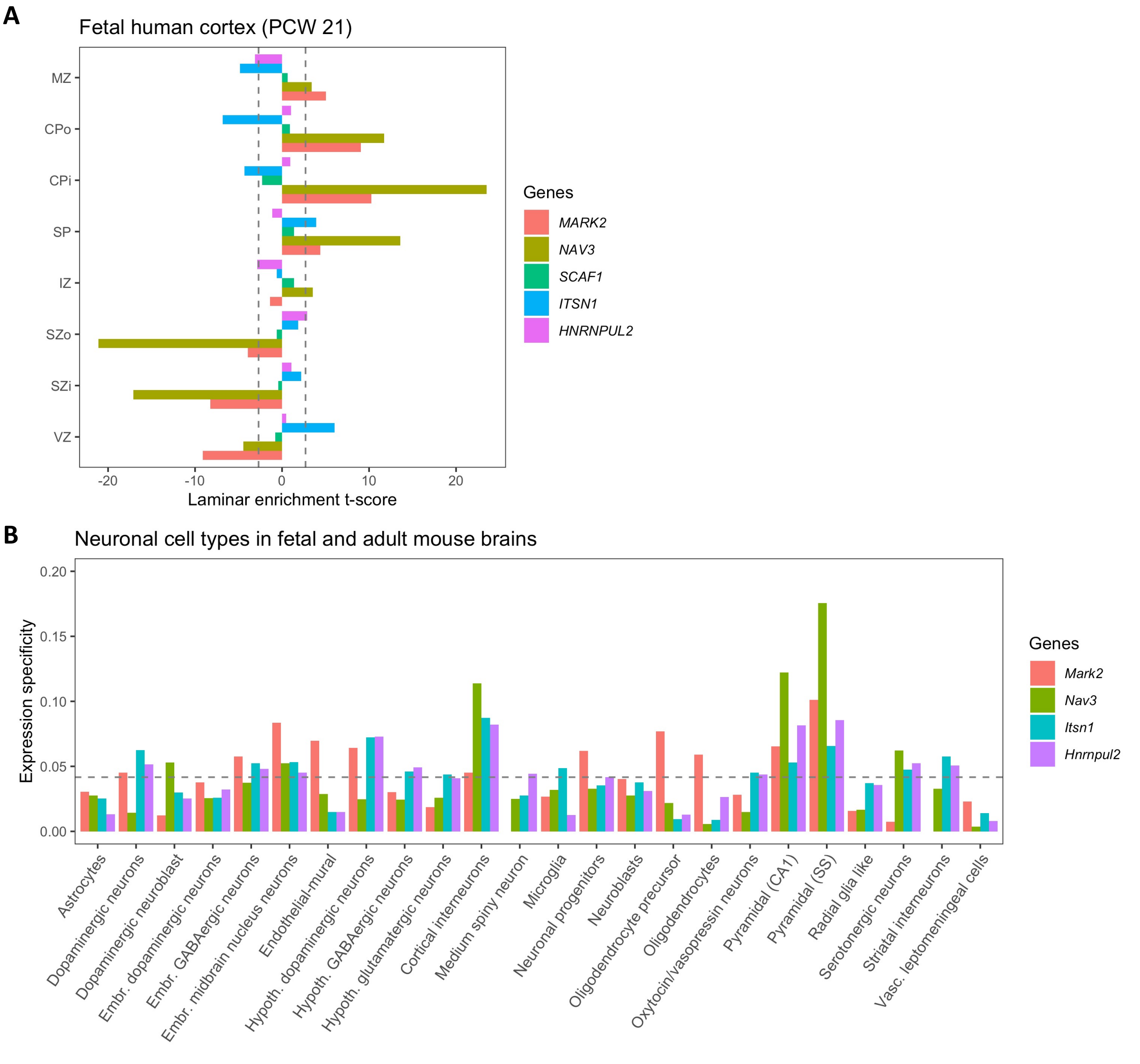


(A) Expression specificities in human fetal cortex laminar at post conceptual week (PCW) 21[49]. The specificity was measured by the t-statistics comparing the expression level in each layer against all other layers. Dashed lines at +/-2.7 corresponds to FDR threshold of 0.01 used in the previous study[49]. Abbreviations: marginal zone (MZ), outer/inner cortical plate (CPo/CPi), subplate (SP), intermediate zone (IZ), outer/inner

subventricular zone (SZo/SZi), ventricular zone (VZ). (B) Expression specificities in neuronal cell types inferred from single cell RNA-seq data of fetal and adult mouse brains[50]. Cell type specificity was measured by mean expression level in one cell type over the summation of mean expression level across all cell types. Dashed line corresponds to uniform expression over 24 cell types. Human genes are mapped to their mouse orthologs. Single-cell expression data for SCAF1 is not available.

## Supplementary Figure S11: Calculated cognitive impairment and sex ratio in individuals with ASD in SPARK

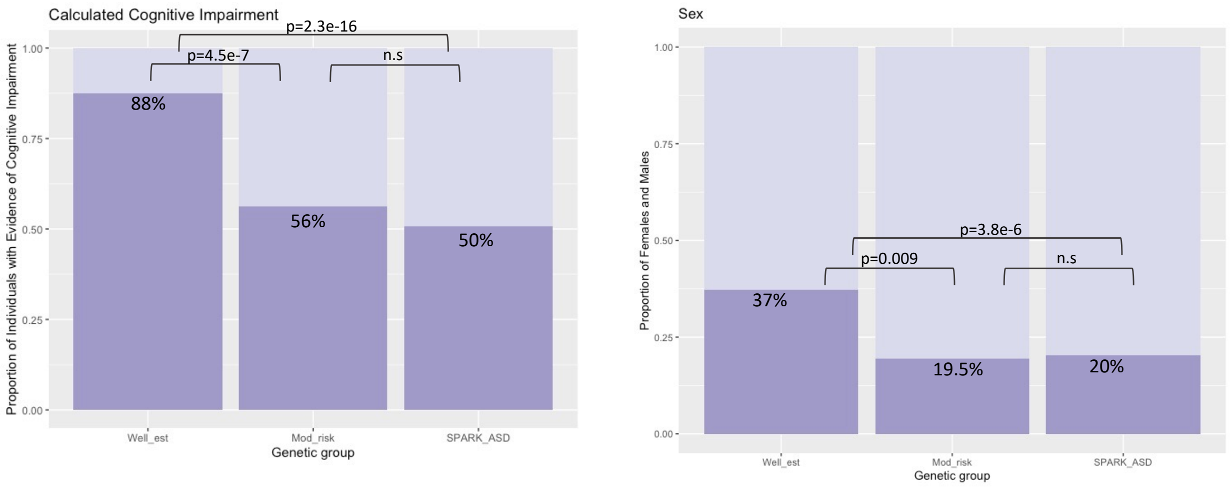


Left panel: the proportion of individuals with ASD with evidence of cognitive impairment is shown in dark purple and the proportion of individuals without evidence of cognitive impairment is shown in light purple. The proportion of in individuals with evidence of cognitive impairment (n=129) with HC LoF variants in well-established, highly-penetrant ASD risk genes (*CHD8, SCN2A, ADNP, FOXP1, SHANK3*) is significantly higher than 8,731 offspring with ASD in SPARK individuals (p=2.3e-16, chi-squared test), although the proportion of individuals (n=87) with LoF. Right panel: the proportion of individuals that are female is shown in dark purple and male is shown in light purple. The proportion of males to females in SPARK (n = 8,731) is 4:1 and is similar in 87 individuals with LoF variants in novel, moderate ASD risk genes. As previously reported, individuals with LoF variants in well-established ASD risk genes show an enrichment of females.

# Supplementary Figure S12: Distribution of different types of LoF variants in known ASD genes enriched by de novo variants (DNVs) and comparison with population controls

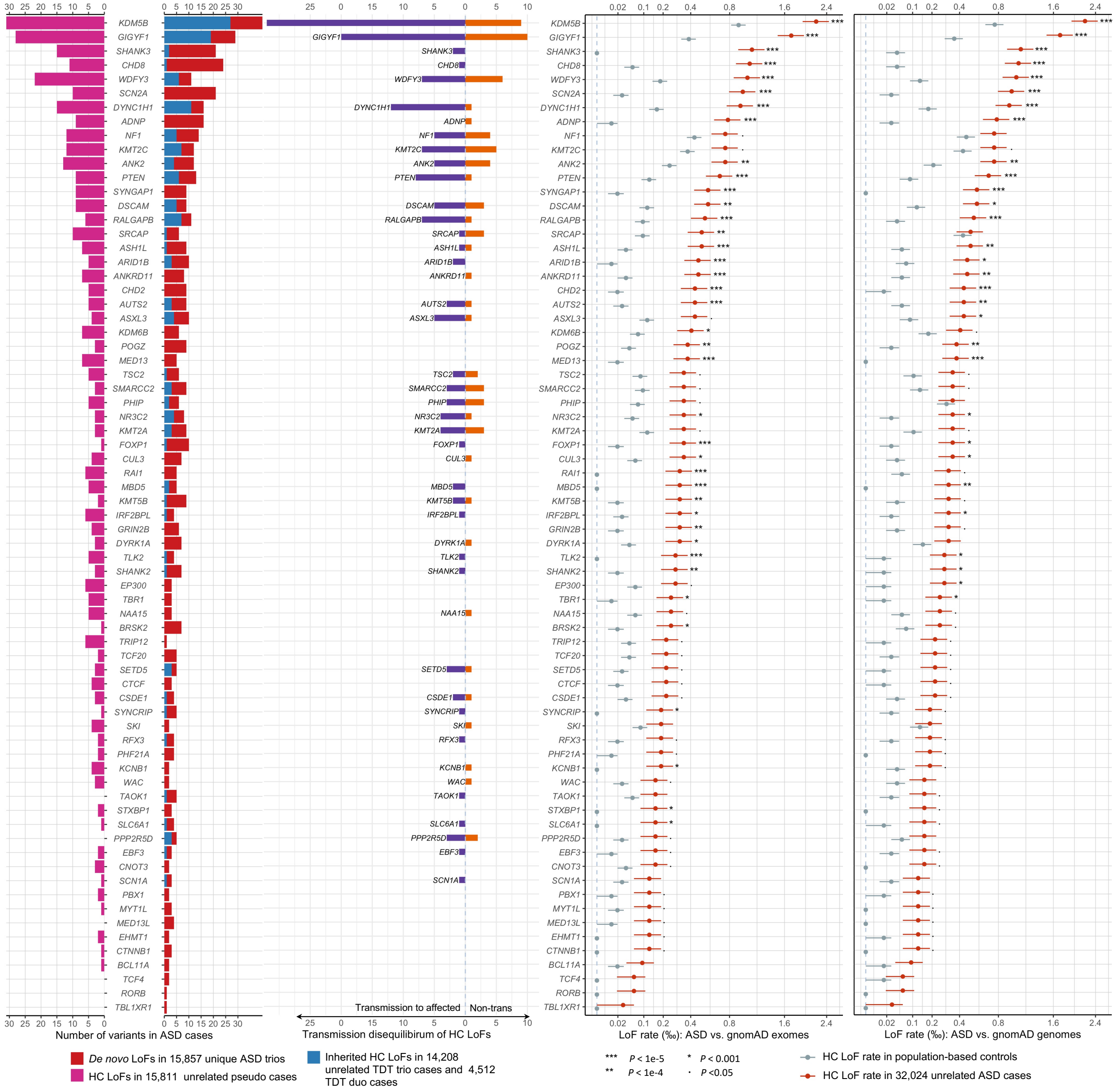


From left to right: pyramid plot summarizing the number of de novo and inherited HC LoFs in family-based samples, HC LoFs in unrelated cases; bar plot of transmission vs.

non-transmission for rare, inherited HC LoFs identified in unaffected parents; and comparing HC LoF rate in cases with two population controls (data are presented as mean values +/- standard errors as error bars). The plot shows 71 known ASD/NDD genes that are in top 30% gnomAD LOEUF and have p-value for enrichment of all DNVs <1e-4 in 23,053 ASD trios. Poisson test was used to compute the p values for comparing the LoF rate between unrelated ASD cases and gnomAD exomes or TopMed controls.

## Supplementary Figure S13: Empirical relationship between estimated relative risk to ASD and estimated selection coefficient

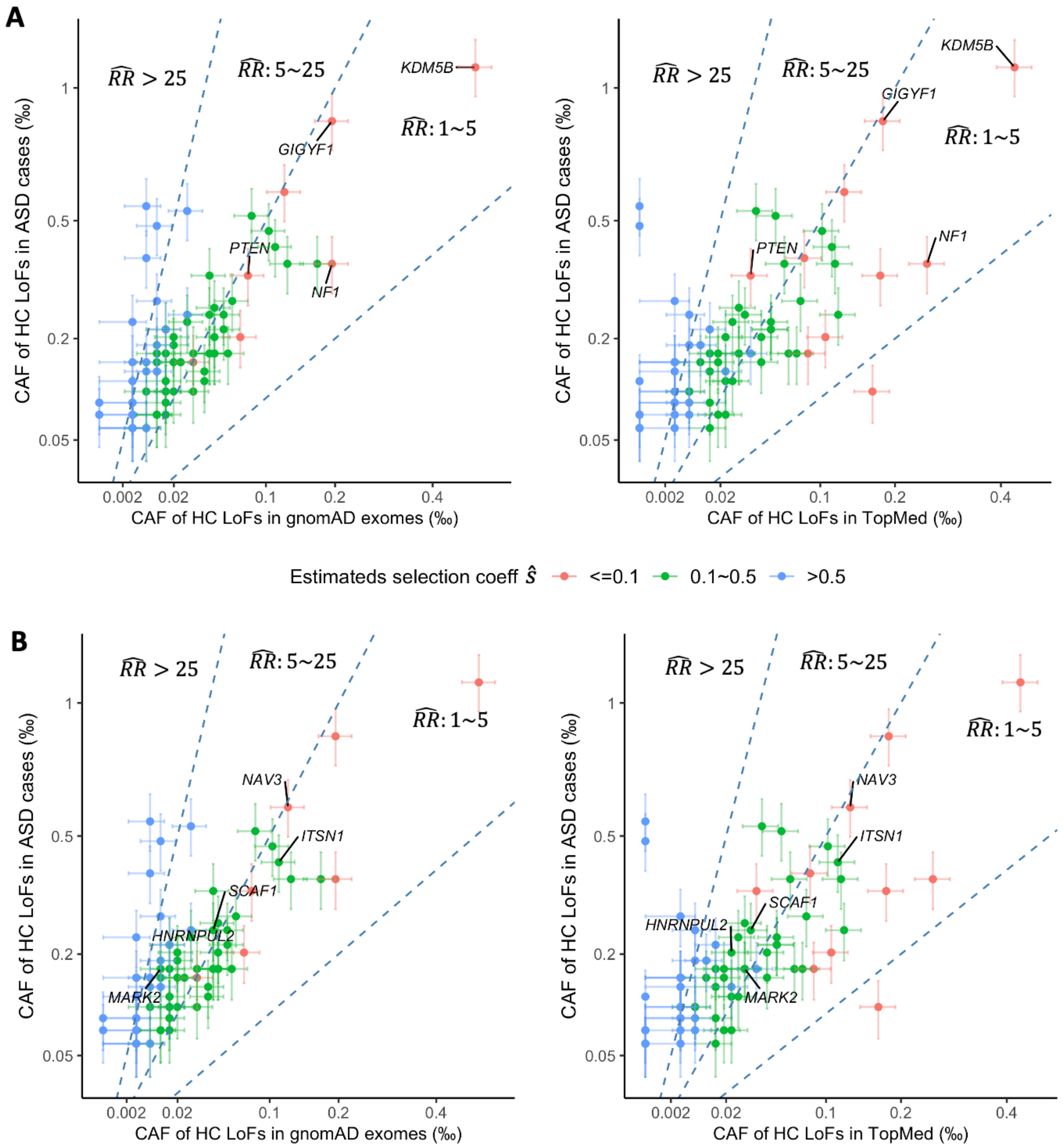

Empirical relationship between estimated relative risk ($\widehat{RR}$) to ASD and estimated selection coefficient ($\hat{s}$). We selected 66 known ASD/NDD genes from top 30% gnomAD LOEUF that have ASD DenovoWEST P-value<1e-4 and LoF mega-analysis P-value<0.05, and also included five novel ASD genes identified from the current study. Panel A highlights four known ASD genes (*PTEN*, *NF1*, *GIGYF1* and *KDM5B*), while Panel B highlights five novel ASD genes (*NAV3*, *ITSN1*, *SCAF1*, *HNRNPUL2* and *MARK2*). Cumulative allele frequencies (CAFs) of HC LoFs were estimated from 32,024 unrelated ASD cases, and two panels of population-based controls from gnomAD and TopMed with sample size 76,000~132,000. Three dashed lines demarcate the quadrant into areas of different estimated relative risks ($\widehat{RR}$: <1, 1~5, 5~25, and >25). Genes with higher effect size to ASD (larger $\widehat{RR}$) are under stronger selective pressure (higher $\hat{s}$).

# Supplementary Figure S14: Burden of de novo and inherited LoFs in genes with high and low LoF mutation rates

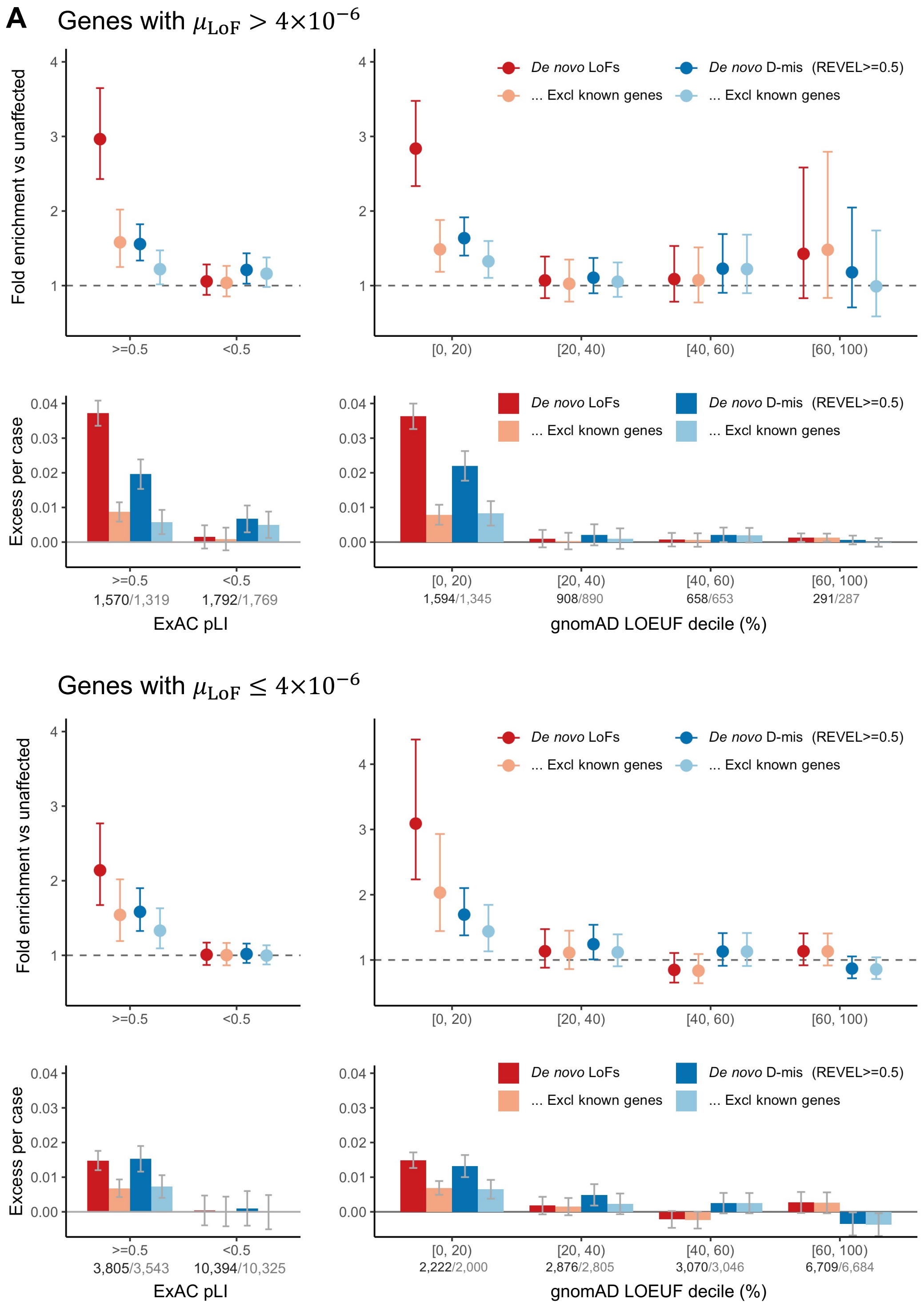

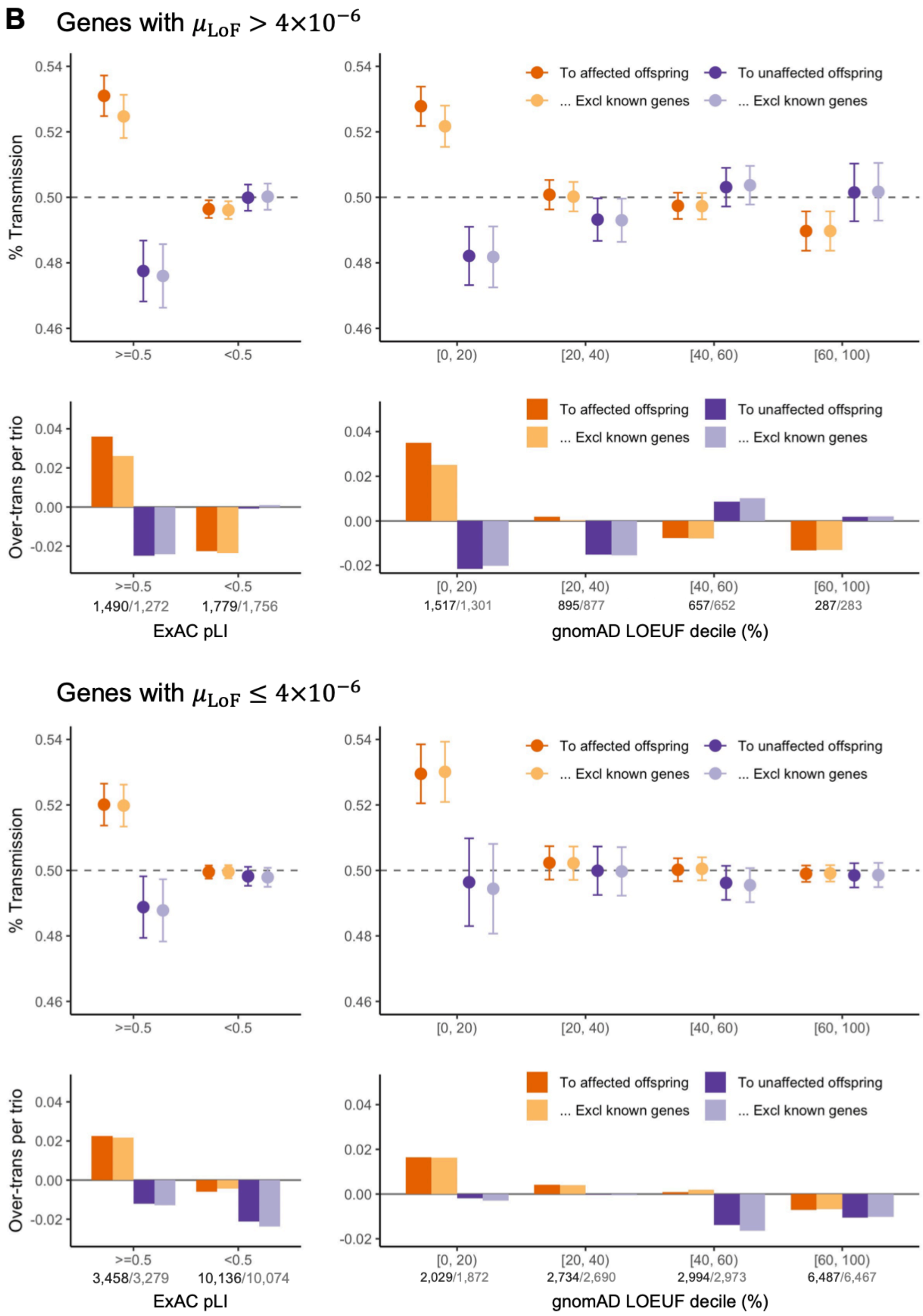
B
Genes with $\mu_{\mathrm{LoF}} > 4\times10^{-6}$
% Transmission
Over-trans per trio
To affected offspring
... Excl known genes
To unaffected offspring
... Excl known genes
>=0.5
<0.5
1,490/1,272
1,779/1,756
ExAC pLI
[0, 20)
[20, 40)
[40, 60)
[60, 100)
1,517/1,301
895/877
657/652
287/283
gnomAD LOEUF decile (%)
Genes with $\mu_{\mathrm{LoF}} \leq 4\times10^{-6}$
3,458/3,279
10,136/10,074
2,029/1,872
2,734/2,690
2,994/2,973
6,487/6,467

(A) The burden of de novo variants among genes with high mutation rate (upper panel) and low mutation rate (lower panel) was evaluated by rate ratio and rate difference between 16,877 ASD and 5,764 unaffected trios. The number of genes before and after removing known genes in each constraint bin was shown below the axis label. (B) Burden of inherited LoFs with high mutation rate (upper panel) and low mutation rate (lower panel) was evaluated by looking at the proportion of rare LoFs in 20,491 unaffected parents that are transmitted to affected offspring in 9,504 trios and 2,966 duos and can be quantified as over-transmission of LoFs per ASD trio. As a comparison, we also show the transmission disequilibrium pattern to unaffected offspring in 5,110 trios and 129 duos. Analysis was restricted to autosomal genes and repeated after removing known ASD/NDD genes (number of genes in each constrained bin before and after removing known genes is shown below the axis label). Data are presented as mean values +/- standard errors as error bars in A and B.

# Supplementary Figure S15: Summary of final DNV call sets for SPARK and SSC discovery samples

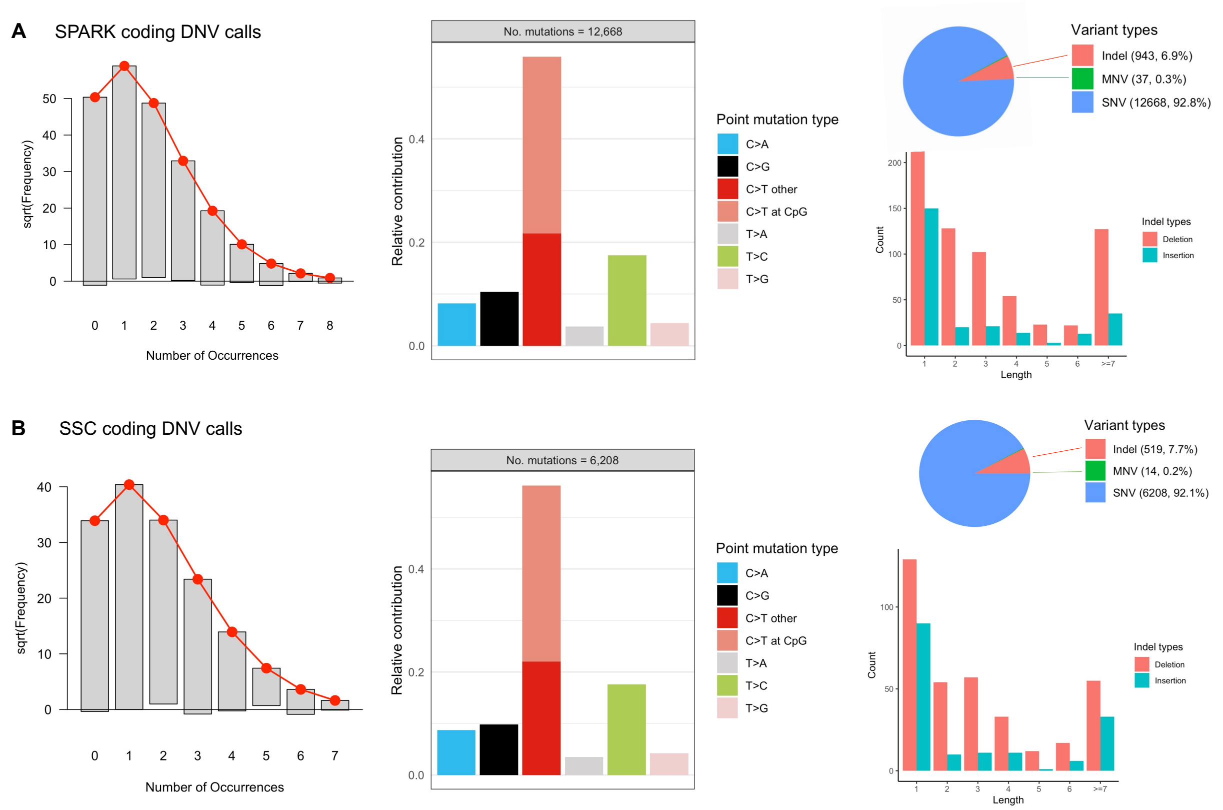


From left to right: identified coding DNVs per trio and fitted Poisson distribution, SNV mutation spectrum, indel length distribution and relative proportion of indels and SNVs. Panel A shows the DNV calls in SPARK, and Panel B shows the DNV calls in SSC.

# Supplementary Figure S16: Evidence of post-zygotic mosaicisms in the final DNV call set

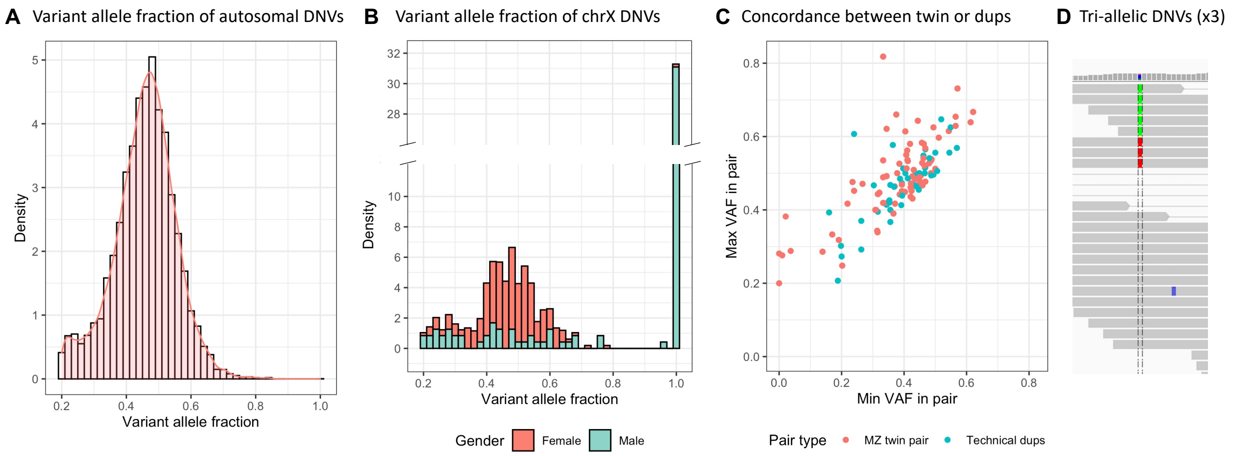


(A) Distribution of variant allele fractions (VAF) of autosomal DNVs shows a small peak at low VAF end. (B) Heterozygous non-PAR chrX DNVs were identified in males. (C) For samples with technical duplicate or MZ twin, VAFs are highly correlated between duplicate or twin pairs. But a small number DNVs with VAF between 0.2 and 0.4 were detected only in one of twin pairs. (D) A small number of DNVs are tri-allelic due to a second post-zygotic mutation.

# Supplementary Figure S17: Rare variant workflow and QC strategy

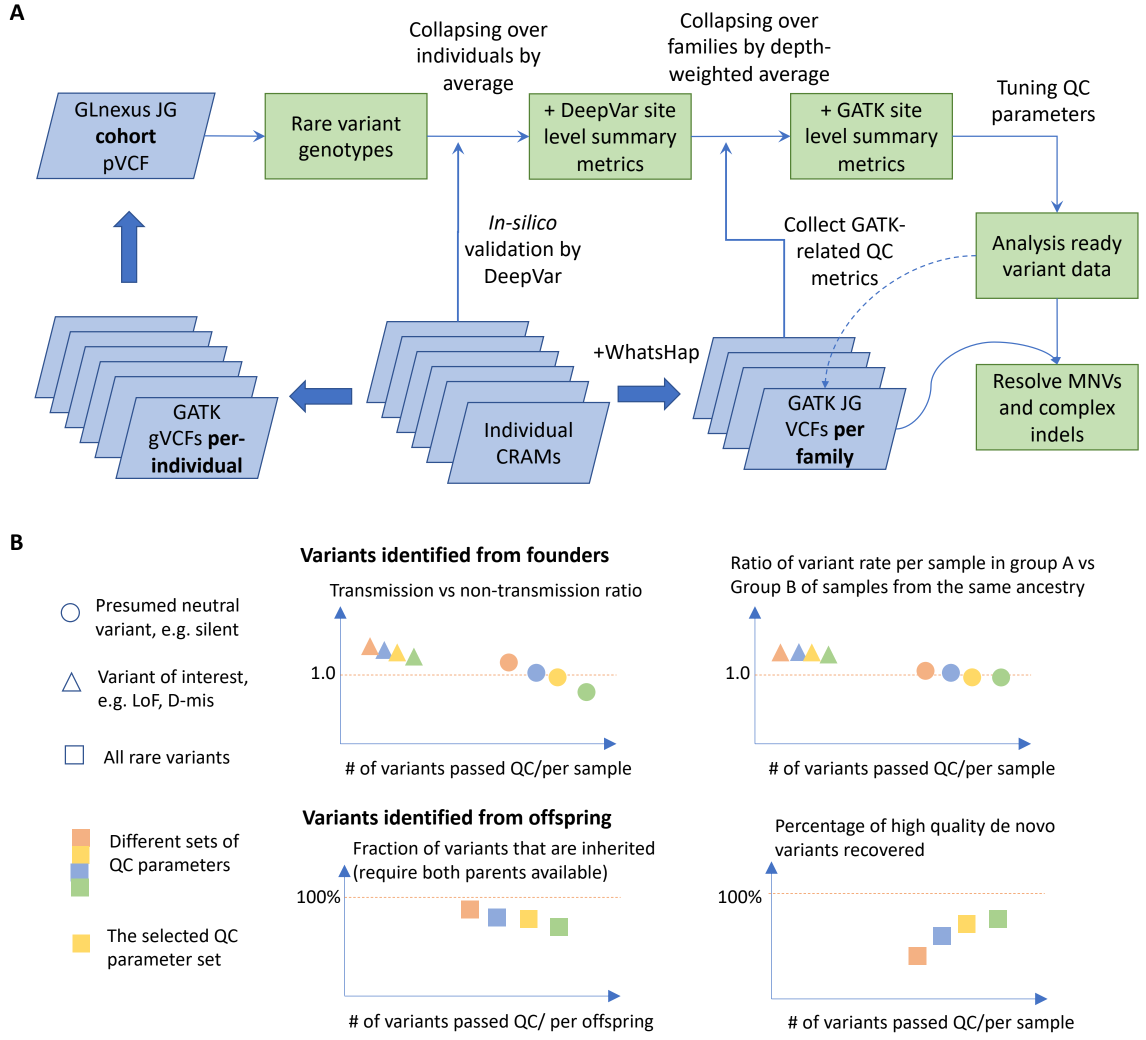


Variant site level QC filters were calibrated using familial transmission information, assuming that false positive calls are more likely to show Mendelian inheritance error. Briefly, we first applied a baseline site level filter that favors high sensitivity, then optimized thresholds for filters with additional QC metrics. The selected QC metrics were reviewed first to determine a small number of optional thresholds. Then the final set of QC parameters were optimized from a grid search over the combinations of available thresholds such that: 1. presumed neutral variants identified from parents

(silent variants or variants in non-constrained genes) shows equal transmission and non-transmission to offspring; 2. rates of neutral variants are similar in different sample groups from the same population ancestry; 3. vast majority variants identified in trio offspring are inherited from parents. In case when multiple sets of QC thresholds give similar results, priority will be given to the set that also recovers maximum number of DNV calls in trio offspring. The optimized filtering parameters were used in final QC filters to generate analysis-ready variants.

## Supplementary Figure S18: Comparison of inhouse DenovoWEST results on NDD trios with published results

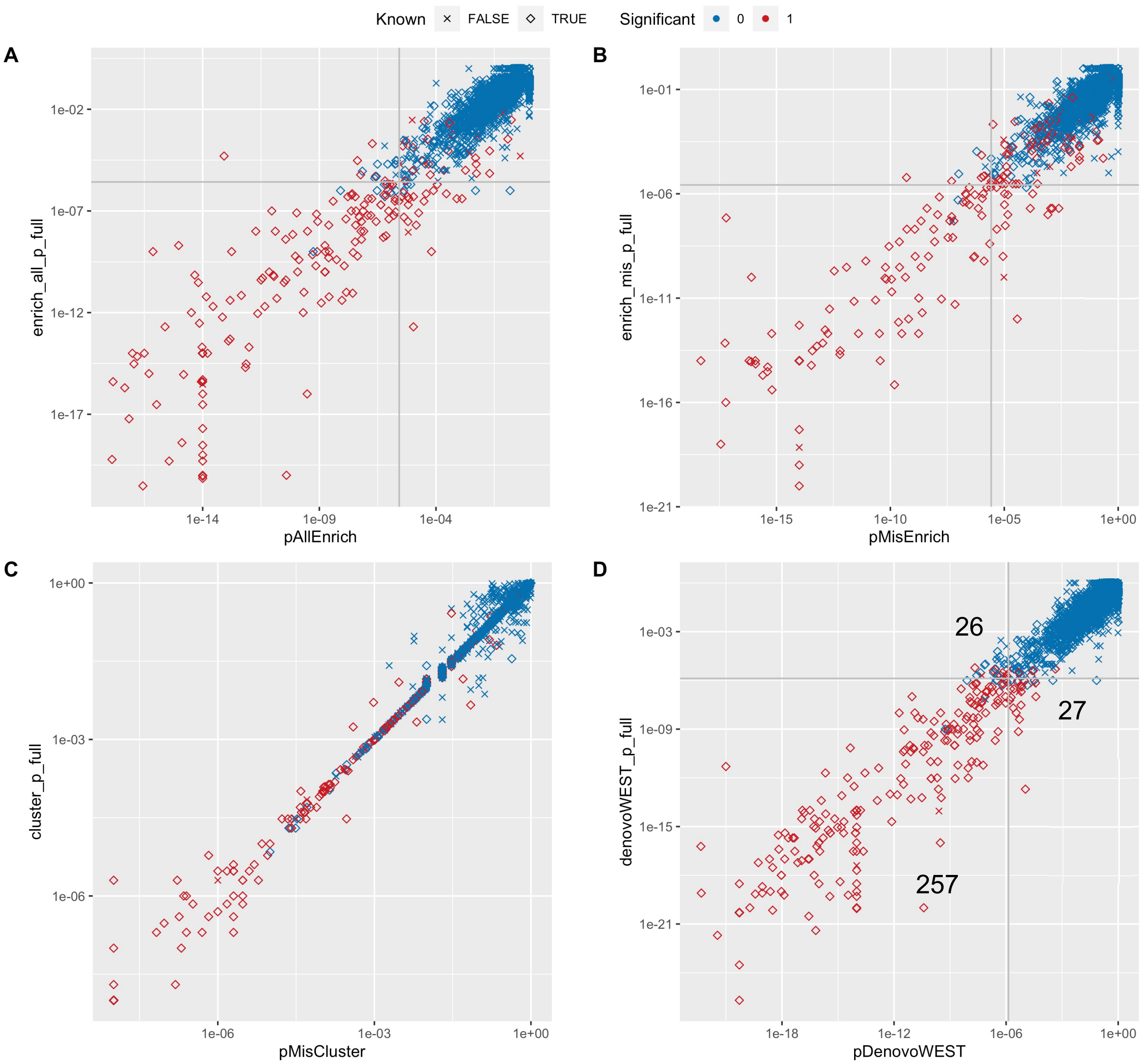


We reannotated DNVs of 31,058 NDD trios from the previous study2 and tested enrichment of DNVs in each coding gene by inhouse implementation of DenovoWEST. For each gene, four different p-values were generated and compared: (A) pAllEnrich: one-sided p-value for enrichment of all DNVs; (B) pMisEnrich: one-sided p-value for

enrichment of missense DNVs; (C) pMisComb: combined p-value for enrichment and clustering of missense DNVs; (D) pDenovowEST: the final DenovoWEST p-value as the minimum of pAllEnrich and pMisComb. P-values from the reanalysis are shown on X-axes and compared with the published p-values on y-axes. Known developmental disease genes included in DDG2P58 (2020-02) are shown in diamond shape, and genes declared exome-wide significant in the previous study was highlighted in red.

# Supplementary Figure S19: Illustration of pseudo cases and contributing sample sizes in different types of pedigrees

**Nuclear families**

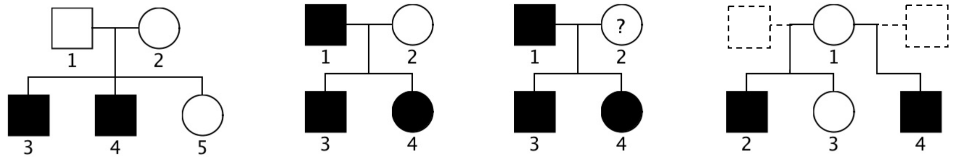


| | | | | |
|---|---|---|---|---|
| **De novo** | Two affected trios (3,4) | Two affected trios (3,4) | Two affected trios (3,4) | 0 |
| **TDT** | Two affected trios (3, 4) | Two affected duos (3,4) | 0: neither parent is unaffected | Two affected duo cases (2,4). |
| **Pseudo cases** | 0 | One affected parent (1) and 2 half affected offspring (3,4; after removing maternal alleles and de novo variants) | One affected parent (1), two full affected offspring (3,4). | Two half affected offspring (2,4; after removing maternal alleles) |

**Multi-generation families**

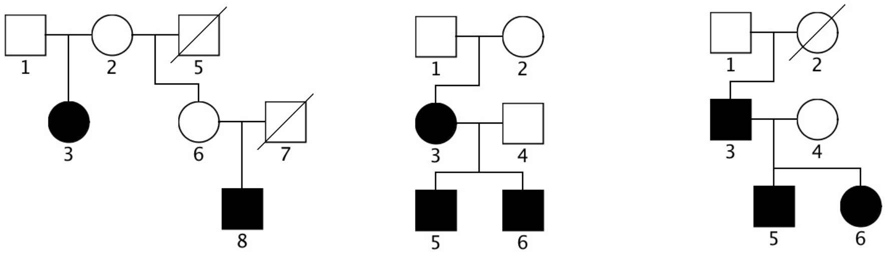


| | | | |
|---|---|---|---|
| **De novo** | Two affected trios (3,8) | Three affected trios (3,5,6) | One affected trio (5) |
| **TDT** | One affected trio (3), one affected duo (8) | One affected trio (3), two affected duos (5,6) | Three affected duos (3,5,6) |
| **Pseudo cases** | One half affected offspring (8; after removing maternal alleles) | 0: For two TDT duos (5,6), alleles not transmitted from unaffected father (4) have been analyzed in affected mother (3) and in their de novo variants. | Two ¼ affected offspring (5,6; after removing maternal alleles, de novo variants, and grand-parent alleles) |

Pseudo cases in family-based samples were created from cases to include variant genotypes that were not used by de novo or TDT analysis. Algorithms used create pseudo cases and determine their contributing sample sizes are described in Methods section.

# Supplementary Figure S20: Workflow for variant filtering on rare inherited LoFs

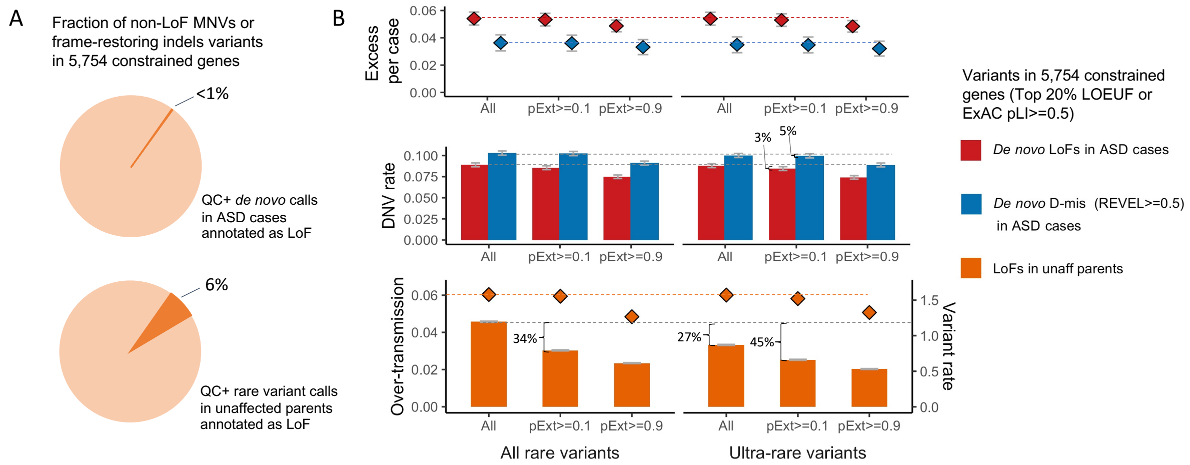


Standing LoFs are notoriously fraught with variant calling artefacts, low confidence LoFs that escape nonsense mediated decay or do not affect splicing[24-26], or LoFs that only affect transcripts that have low expression in disease relevant tissues[27]. In constrained genes, we found about 6% QC passed variant calls initially annotated as LoFs are part of non-LoF MNV or frame-restoring indels, in contrast to <1% in dnLoFs (A). To prioritize high confidence standing LoFs, we applied of LOFTEE/pExt and allele frequency filters. Using pExt>=0.1 in developing brain removes more than 1/3 of LoFs without changing the over-transmission rate to affected offspring. Further applying ultra-rare allele frequency filter (allele frequency<1.5e-4 or singleton in cohort and <5e-5 in populations) removes additional 11% standing LoFs with minimal changes to over-transmission rate. Together, close to half of standing LoF variants are removed that does not contribute to ASD in offspring. Although further increasing pExt threshold to 0.9 will reduce over-transmission rate, it is likely that optimal pExt threshold is gene-

specific and we may underestimate fraction of standing LoFs that does not contribute to ASD. In comparison, the same set of filters only removes 3% dnLoFs and 5% dnDmis in ASD, 3% dnLoFs and 2% dnDmis in other NDD with minimal changes to rate difference between affected and control trios (B).

# Supplementary Figure S21: SPARK sample QC: relatedness check and sex validation

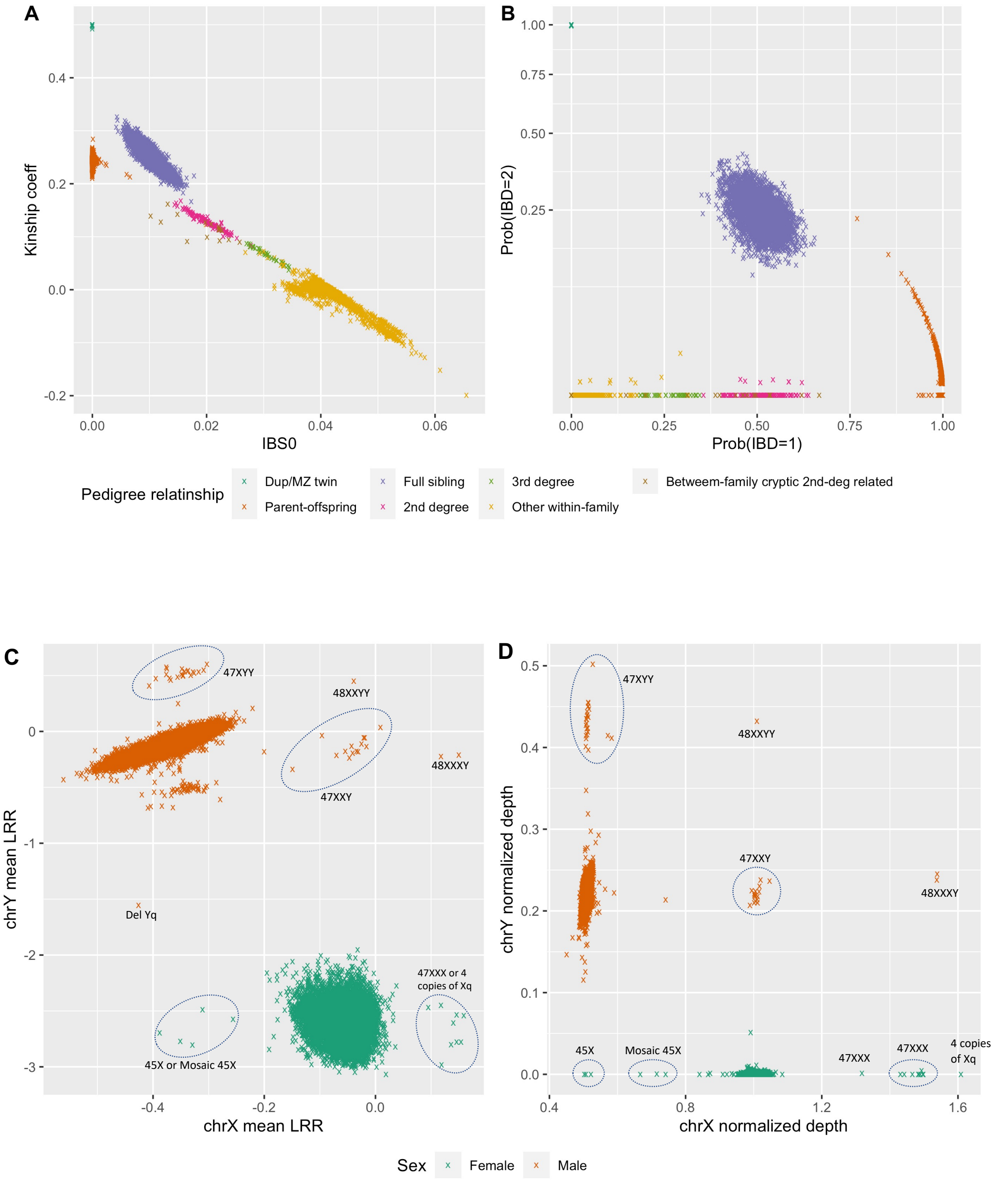

(A-B) Known pedigree relationships are verified by estimated kinship coefficients, proportion of SNPs with zero alleles shared identity by state (IBS0), and probabilities of 1 or 2 copies of chromosomes shared identity by descent (Prob(IBD=1) and Prob(IBD=2)). (C-D) Sample sexes are verified by log-R ratio (LRR) signals and normalized read depth of sex chromosomes. Samples with sex chromosome aneuploidies are highlighted.

# Supplementary Figure S22: SPARK principal component analysis (PCA) and ancestry inference

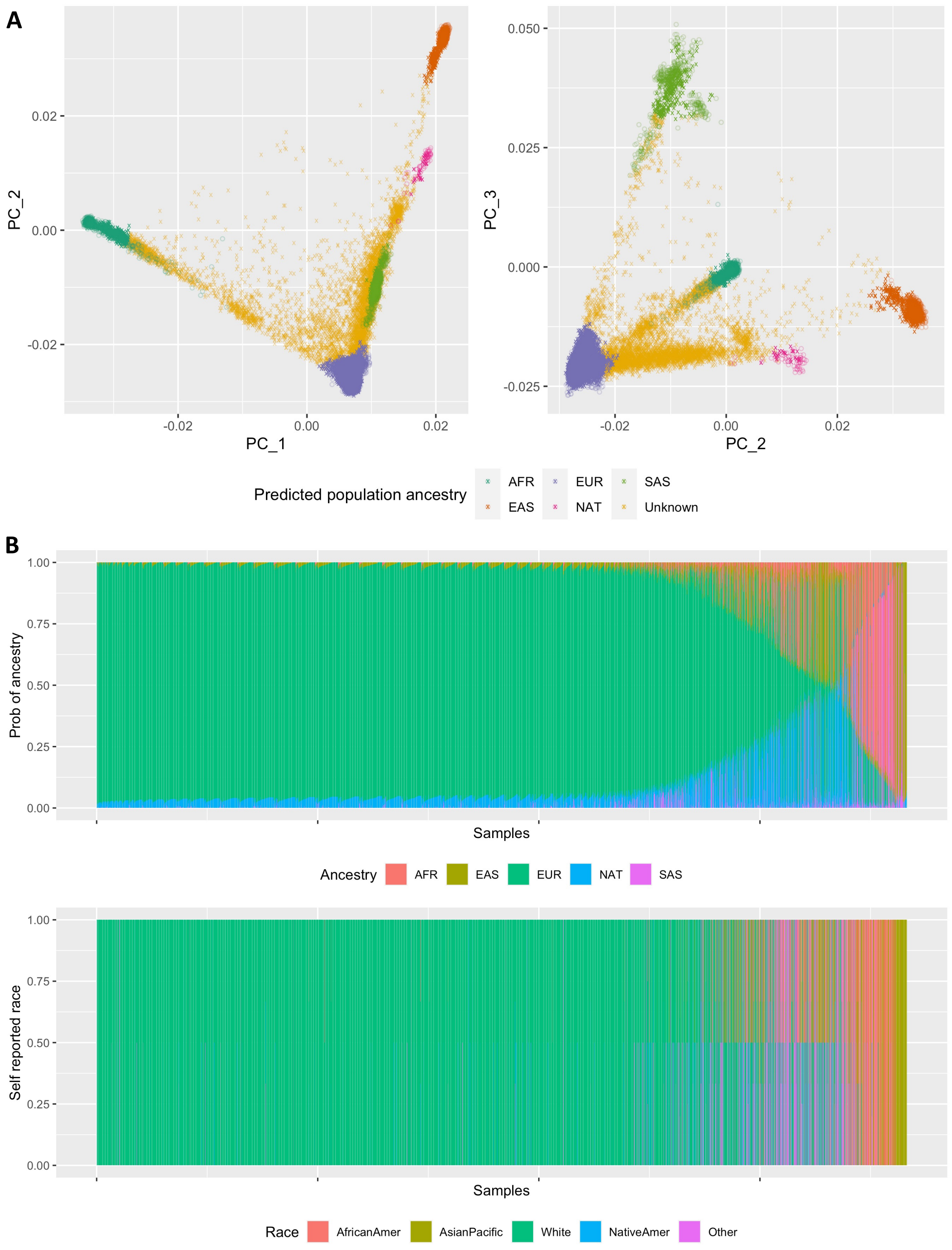

(A) PCA was first performed on samples from five reference populations including 650 Africans (AFR), 504 east Asians (EAS), 503 Europeans (EUR) and 486 south Asians (SAS) from 1000 Genomes Project and 48 native Americans (NAT) from HGDP-CEPH panel. SNP genotypes of 28,649 SPARK samples in the discovery cohort were then projected onto the principal axes of defined by the reference populations. The top three principal axes are shown: circle points are samples from reference populations, cross points are SPARK samples. The projected coordinates at top four axes are transformed to the probabilities of population ancestries using SNPweights method25. Sample is predicted to originate from one reference population if the corresponding probability >=0.8. Samples whose origin cannot be classified by the above criteria are labeled as unknown. (B) Comparing self-reported race(s) and inferred probabilities of population ancestries. For 7,176 offspring cases, self-reported race(s) are available, which include one or more of the following: black or African American (AfricanAmer), Asian or Native Hawaiian or other Pacific Islander (AsianPacific), White, American Indian or Alaska Native (NativeAmer), and Other. Inferred probability of five reference populations for each individual sum up to 1 and are visualized using a stacked bar plot. Individuals are ordered by the probability of EUR, AFR, ASI and NAT. Self-reported races for the same set of individuals are also visualized below as a bar plot. For individuals with multiple self-reported races, multiple races are shown as sub-bars with equal length. There is a general concordance of individuals with self-reported white and Asian with predicted EUR and ASI ancestries. Individuals with self-reported African American or native American are more likely to have recent admixture of EUR, AFR and NAT.

## Supplementary Figure S23: SPARK self-reported cognitive impairment shows stronger correlation with Vineland score than full-scale IQ

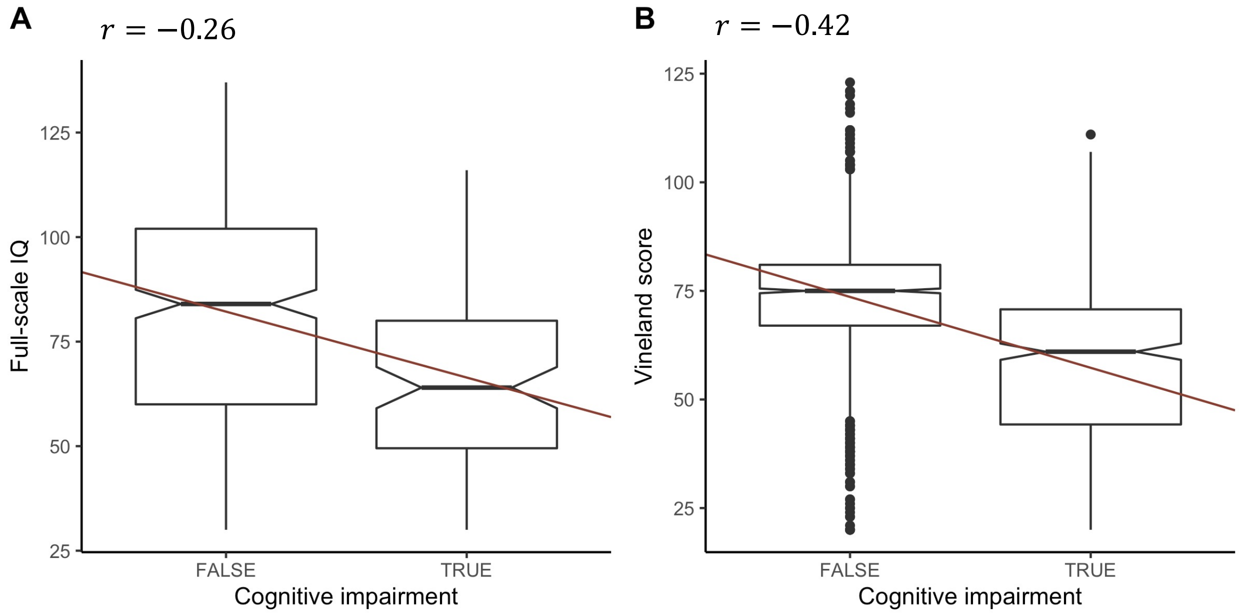


(A) In 478 samples with full-scale IQ, Pearson correlation between IQ and cognitive impairment is -0.26. (B) In 2183 samples with standardized Vineland score, correlation between Vineland scores and cognitive impairment is -0.42. The box plots represent median as center, inter-quartile range (IQR) as bounds of box and the upper whisker extends from the upper bound of box to 1.5*IQR and the lower whisker extends from the lower bound of box to 1.5*IQR. Data beyond the end of the whiskers are outliers and plotted as points.

## Supplementary Figure S24: Comparing phenotypes of samples from simplex and multiplex families in SPARK cohort

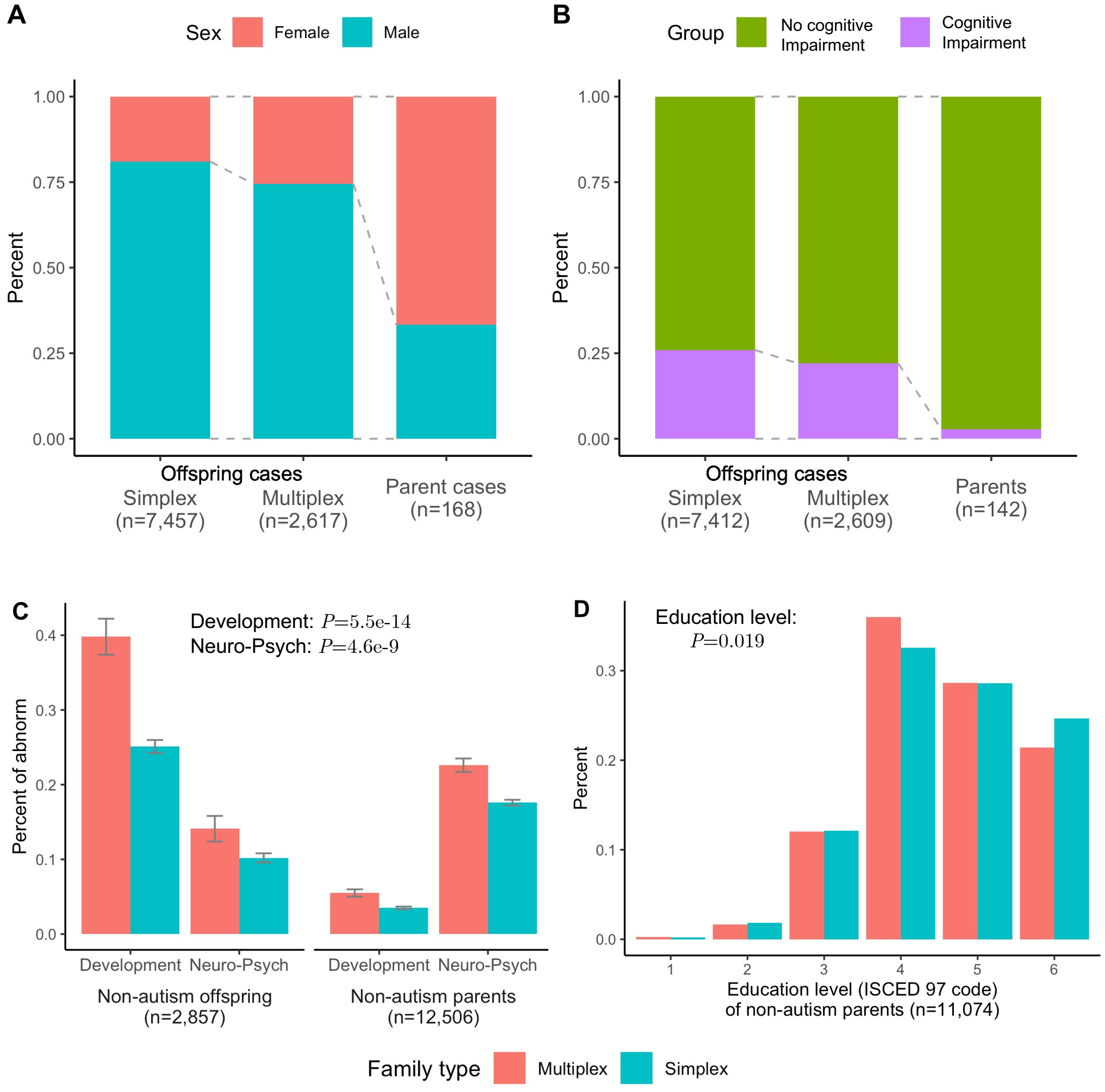


Multiplex families are defined as families with at least one pair of affected first degree relatives at recruitment or by self-report, all other families are simplex. (A) There is more

female ASD cases in multiplex families, especially among affected parents. (B) Parent cases are also less likely to have cognitive impairment. Sample sizes shown below each group are the number of samples with non-missing information. (C) Unaffected family members in multiplex families are more likely to have other developmental or neuro-psychiatric issues than simplex families. Developmental issues include structural birth defects, learning or language disability, motor delays, social communication problems, etc. Neuro-psychiatric issues include seizure, schizophrenia or schizoaffective disorder, bipolar disorder, Tourette syndrome, etc. The association with family history was evaluated by logistic regression adjusting sex and role in the family. Data are presented as mean values +/- standard errors as error bars. (D) Unaffected parents in multiplex families also have lower education attainment than unaffected parents in simplex families (P=0.019, by linear regression adjusting sex). Education level were coded by International Standard Classification of Education (1997).

# Reference

1. Li, H. Aligning sequence reads, clone sequences and assembly contigs with BWA-MEM. *arXiv* (2013).
2. Fritz, M.H.Y., Leinonen, R., Cochrane, G. & Birney, E. Efficient storage of high throughput DNA sequencing data using reference-based compression. *Genome Res* **21**, 734-740 (2011).
3. Institute, P.T.-B.B. https://broadinstitute.github.io/picard/.
4. Pedersen, B.S. & Quinlan, A.R. Mosdepth: quick coverage calculation for genomes and exomes. *Bioinformatics* **34**, 867-868 (2018).
5. Jun, G. *et al.* Detecting and estimating contamination of human DNA samples in sequencing and array-based genotype data. *Am J Hum Genet* **91**, 839-48 (2012).
6. Poplin, R. *et al.* Scaling accurate genetic variant discovery to tens of thousands of samples. *bioRxiv*, 201178 (2018).
7. GitHub - Genomicsplc/wecall: Fast, accurate and simple to use command line tool for variant detection in NGS data. https://github.com/Genomicsplc/wecall.
8. Poplin, R. *et al.* A universal SNP and small-indel variant caller using deep neural networks. *Nat Biotechnol* **36**, 983-987 (2018).
9. Lin, M.F. *et al.* GLnexus: joint variant calling for large cohort sequencing. *bioRxiv*, 343970 (2018).
10. Garrison, E. & Marth, G. Haplotype-based variant detection from short-read sequencing. *arXiv* (2012).
11. Martin, M. *et al.* WhatsHap: fast and accurate read-based phasing. *bioRxiv*, 085050 (2016).
12. Tan, A., Abecasis, G.R. & Kang, H.M. Unified representation of genetic variants. *Bioinformatics* **31**, 2202-4 (2015).
13. Frankish, A. *et al.* GENCODE reference annotation for the human and mouse genomes. *Nucleic Acids Res* **47**, D766-d773 (2019).
14. Autism Spectrum Disorders Working Group of The Psychiatric Genomics, C. Meta-analysis of GWAS of over 16,000 individuals with autism spectrum disorder highlights a novel locus at 10q24.32 and a significant overlap with schizophrenia. *Molecular autism* **8**, 21-21 (2017).
15. Cummings, B.B. *et al.* Transcript expression-aware annotation improves rare variant interpretation. *Nature* **581**, 452-458 (2020).
16. Lindsay, S.J. *et al.* HDBR Expression: A Unique Resource for Global and Individual Gene Expression Studies during Early Human Brain Development. *Front Neuroanat* **10**, 86 (2016).
17. Ioannidis, N.M. *et al.* REVEL: An Ensemble Method for Predicting the Pathogenicity of Rare Missense Variants. *Am J Hum Genet* **99**, 877-885 (2016).
18. Kircher, M. *et al.* A general framework for estimating the relative pathogenicity of human genetic variants. *Nat Genet* **46**, 310-5 (2014).
19. Samocha, K.E. *et al.* Regional missense constraint improves variant deleteriousness prediction. *bioRxiv*, 148353 (2017).
20. Sundaram, L. *et al.* Predicting the clinical impact of human mutation with deep neural networks. *Nat Genet* **50**, 1161-1170 (2018).
21. Karczewski, K.J. *et al.* The mutational constraint spectrum quantified from variation in 141,456 humans. *Nature* **581**, 434-443 (2020).
22. Lek, M. *et al.* Analysis of protein-coding genetic variation in 60,706 humans. *Nature* **536**, 285-91 (2016).

23. Jaganathan, K. *et al.* Predicting Splicing from Primary Sequence with Deep Learning. *Cell* **176**, 535-548 e24 (2019).
24. Karczewski, K.J. *et al.* The mutational constraint spectrum quantified from variation in 141,456 humans. *Nature* **581**, 434-443 (2020).
25. MacArthur, D.G. *et al.* A systematic survey of loss-of-function variants in human protein-coding genes. *Science* **335**, 823-828 (2012).
26. Lek, M. *et al.* Analysis of protein-coding genetic variation in 60,706 humans. *Nature* **536**, 285-291 (2016).
27. Cummings, B.B. *et al.* Transcript expression-aware annotation improves rare variant interpretation. *Nature* **581**, 452-458 (2020).
28. Chen, W.M., Manichaikul, A. & Rich, S.S. A generalized family-based association test for dichotomous traits. *Am J Hum Genet* **85**, 364-76 (2009).
29. Staples, J. *et al.* PRIMUS: rapid reconstruction of pedigrees from genome-wide estimates of identity by descent. *Am J Hum Genet* **95**, 553-64 (2014).
30. Genomes Project, C. *et al.* A global reference for human genetic variation. *Nature* **526**, 68-74 (2015).
31. Li, J.Z. *et al.* Worldwide human relationships inferred from genome-wide patterns of variation. *Science* **319**, 1100-4 (2008).
32. Bergstrom, A. *et al.* Insights into human genetic variation and population history from 929 diverse genomes. *Science* **367**(2020).
33. Patterson, N., Price, A.L. & Reich, D. Population structure and eigenanalysis. *PLoS Genet* **2**, e190 (2006).
34. Chen, C.Y. *et al.* Improved ancestry inference using weights from external reference panels. *Bioinformatics* **29**, 1399-406 (2013).
35. Bishop, S.L., Farmer, C. & Thurm, A. Measurement of nonverbal IQ in autism spectrum disorder: scores in young adulthood compared to early childhood. *Journal of autism and developmental disorders* **45**, 966-974 (2015).
36. Munson, J. *et al.* Evidence for latent classes of IQ in young children with autism spectrum disorder. *American journal of mental retardation : AJMR* **113**, 439-452 (2008).
37. Shu, C., Snyder, L.G., Shen, Y., Chung, W.K. & Consortium, o.b.o.t.S. Imputing cognitive impairment in SPARK, a large autism cohort. *medRxiv*, 2021.08.25.21262613 (2021).
38. Packer, J.S. *et al.* CLAMMS: a scalable algorithm for calling common and rare copy number variants from exome sequencing data. *Bioinformatics* **32**, 133-5 (2016).
39. Koehler, R., Issac, H., Cloonan, N. & Grimmond, S.M. The uniqueome: a mappability resource for short-tag sequencing. *Bioinformatics* **27**, 272-4 (2011).
40. Fischbach, G.D. & Lord, C. The Simons Simplex Collection: a resource for identification of autism genetic risk factors. *Neuron* **68**, 192-5 (2010).
41. Iossifov, I. *et al.* The contribution of de novo coding mutations to autism spectrum disorder. *Nature* **515**, 216-21 (2014).
42. Krumm, N. *et al.* Excess of rare, inherited truncating mutations in autism. *Nat Genet* **47**, 582-8 (2015).
43. An, J.Y. *et al.* Genome-wide de novo risk score implicates promoter variation in autism spectrum disorder. *Science* **362**(2018).
44. Werling, D.M. *et al.* An analytical framework for whole-genome sequence association studies and its implications for autism spectrum disorder. *Nat Genet* **50**, 727-736 (2018).
45. Buxbaum, J.D. *et al.* The autism sequencing consortium: large-scale, high-throughput sequencing in autism spectrum disorders. *Neuron* **76**, 1052-6 (2012).
46. De Rubeis, S. *et al.* Synaptic, transcriptional and chromatin genes disrupted in autism. *Nature* **515**, 209-15 (2014).

47. Satterstrom, F.K. *et al.* Large-Scale Exome Sequencing Study Implicates Both Developmental and Functional Changes in the Neurobiology of Autism. *Cell* **180**, 568-584 e23 (2020).
48. Yuen, R.K. *et al.* Whole genome sequencing resource identifies 18 new candidate genes for autism spectrum disorder. *Nat Neurosci* **20**, 602-611 (2017).
49. Parikshak, N.N. *et al.* Integrative functional genomic analyses implicate specific molecular pathways and circuits in autism. *Cell* **155**, 1008-1021 (2013).
50. Skene, N.G. *et al.* Genetic identification of brain cell types underlying schizophrenia. *Nature genetics* **50**, 825-833 (2018).
