# Supplementary material for: Integrating de novo and inherited variants in 42,607 autism cases identifies mutations in new moderate-risk genes
Source: Nat Genet. 2022 Aug 18;54(9):1305–19. doi: 10.1038/s41588-022-01148-2 (PMC9470534; doi:10.1038/s41588-022-01148-2)
Supplement: Supplementary file 2 — Reporting Summary [file 41588_2022_1148_MOESM2_ESM.pdf]

nature portfolio

Corresponding author(s): Chung, W.K.

Last updated by author(s): 2022-April-30

Reporting Summary

Nature Portfolio wishes to improve the reproducibility of the work that we publish. This form provides structure for consistency and transparency in reporting. For further information on Nature Portfolio policies, see our Editorial Policies and the Editorial Policy Checklist.

Statistics

For all statistical analyses, confirm that the following items are present in the figure legend, table legend, main text, or Methods section.

n/a	Confirmed	
☐	☒	The exact sample size (*n*) for each experimental group/condition, given as a discrete number and unit of measurement
☒	☐	A statement on whether measurements were taken from distinct samples or whether the same sample was measured repeatedly
☐	☒	The statistical test(s) used AND whether they are one- or two-sided<br>*Only common tests should be described solely by name; describe more complex techniques in the Methods section.*
☐	☒	A description of all covariates tested
☐	☒	A description of any assumptions or corrections, such as tests of normality and adjustment for multiple comparisons
☐	☒	A full description of the statistical parameters including central tendency (e.g. means) or other basic estimates (e.g. regression coefficient) AND variation (e.g. standard deviation) or associated estimates of uncertainty (e.g. confidence intervals)
☐	☒	For null hypothesis testing, the test statistic (e.g. *F*, *t*, *r*) with confidence intervals, effect sizes, degrees of freedom and *P* value noted<br>*Give P values as exact values whenever suitable.*
☐	☒	For Bayesian analysis, information on the choice of priors and Markov chain Monte Carlo settings
☐	☒	For hierarchical and complex designs, identification of the appropriate level for tests and full reporting of outcomes
☐	☒	Estimates of effect sizes (e.g. Cohen's *d*, Pearson's *r*), indicating how they were calculated

Our web collection on statistics for biologists contains articles on many of the points above.

Software and code

Policy information about availability of computer code

Data collection	All participants were recruited to SPARK under a centralized IRB protocol (Western IRB Protocol #20151664). All participants provided written informed consent to take part in the study. Written informed consent was obtained from all legal guardians or parents for all participants age 18 and younger and all participants age 18 and older who have a legal guardian. Assent was also obtained from dependent participants age 10 and older. Participants with autism were compensated $25-50 depending on other registered family members. Saliva samples were collected from participants and used for DNA extraction. New exome sequencing samples in this study were captured by IDT xGEN research panel and sequenced on Illumina NovaSeq. DNA samples were also genotyped for over 600K SNPs by Infinium Global Screening Array.
Data analysis	All software used in this study is publicly available. Supplementary Table S16 describes software versions and parameter settings used. The code for major figures and analysis under https://github.com/ShenLab/SPARK_Analysis_V1.git. The DOI is 10.5281/zenodo.6646871.

For manuscripts utilizing custom algorithms or software that are central to the research but not yet described in published literature, software must be made available to editors and reviewers. We strongly encourage code deposition in a community repository (e.g. GitHub). See the Nature Portfolio guidelines for submitting code & software for further information.

Data

Policy information about availability of data

All manuscripts must include a data availability statement. This statement should provide the following information, where applicable:

- Accession codes, unique identifiers, or web links for publicly available datasets
- A description of any restrictions on data availability
- For clinical datasets or third party data, please ensure that the statement adheres to our policy

In order to abide by the informed consents that individuals with autism and their family members signed when agreeing to participate in a SFARI cohort (SSC and

SPARK), researchers must be approved by SFARIbase (https://base.sfari.org). To access to SPARK/SFARI data, researchers should
1) Obtain a SFARI base account at https://base.sfari.org, which will require affiliating with an institution. Currently there are 271 institutions around the world that have signed SFARI's RDA and any researcher affiliated with those institutions can apply for SFARI base access.
2) Review the institute's executed Researcher Distribution Agreement (RDA). The standard RDA is here: https://s3.amazonaws.com/sf-web-assets-prod/wp-content/uploads/sites/2/2021/06/15165956/SFARI_RDA.pdf
3) Create a SFARI base project, which includes a title, abstract and an IRB approval or exemption document.
4) Create a SFARI base request. All requests are processed in a timely manner.

The SPARK data is accessible as follows:
SFARI_SPARK_iWES: This includes exome and genotyping data on 70,487 participants, including all people analyzed in this paper plus an additional 11,282 participants.
SFARI_SPARK_WGS_1: This includes whole genome data from 2,629 individuals from 645 families with at least one person with autism.
SFARI_SPARK_WGS_2: This includes whole genome data from 2,365 individuals, from 587 families with at least one person with autism.
SFARI_SPARK_WGS_3: This includes whole genome data from 2,871 individuals, from 803 families with at least one person with autism.
SSC_WES_3: This is whole exome data on the Simons Simplex Collection (SSC) as analyzed and reported by Krumm et al, 2015.
SFARI_SSC_WGS_pilot: This is genomes of 40 families of autism.
SFARI_SSC_WGS_1 and SFARI_SSC_ WGS_2: WGS of the SSC.
SSC Dataset: Phenotypic information on 2,644 simplex autism families.
SPARK Phenotype Dataset V7: This is the current phenotypic dataset on 290,502 SPARK participants, including 111,720 participants with autism.

Field-specific reporting

Please select the one below that is the best fit for your research. If you are not sure, read the appropriate sections before making your selection.

☒ Life sciences ☐ Behavioural & social sciences ☐ Ecological, evolutionary & environmental sciences

For a reference copy of the document with all sections, see nature.com/documents/nr-reporting-summary-flat.pdf

Life sciences study design

All studies must disclose on these points even when the disclosure is negative.

Sample size	The sample size of people with autism is the largest genomic analysis of people with autism to date. The SPARK cohort represents the participants that registered, provided informed consent and provided a DNA sample between 2015-2019.
Data exclusions	We excluded parents with autism and intellectual disability from the TDT analysis.
Replication	A set of 400 genes was prioritized in the first stage of the analysis. These genes were analyzed in the 2nd stage of analysis. The experiments have not been replicated although other papers (Fu et al, in press at Nature Genetics) have performed complementary analyses with consistent results.
Randomization	All SPARK participants reside in the US, speak English and reported whether or not they had a professional diagnosis of autism. Covariates are not relevant to the genetic basis for autism as the group includes a very large number of diverse participants.
Blinding	Participants registered in the study as having autism or not so we were unable to blind the study. All families in SPARK had to have at least one family member with a professional diagnosis of autism.

Reporting for specific materials, systems and methods

We require information from authors about some types of materials, experimental systems and methods used in many studies. Here, indicate whether each material, system or method listed is relevant to your study. If you are not sure if a list item applies to your research, read the appropriate section before selecting a response.

Materials & experimental systems

n/a	Involved in the study
☒	☐ Antibodies
☒	☐ Eukaryotic cell lines
☒	☐ Palaeontology and archaeology
☒	☐ Animals and other organisms
☐	☒ Human research participants
☒	☐ Clinical data
☒	☐ Dual use research of concern

Methods

n/a	Involved in the study
☒	☐ ChIP-seq
☒	☐ Flow cytometry
☒	☐ MRI-based neuroimaging

Human research participants

Policy information about studies involving human research participants

Population characteristics	Any person with a professional diagnosis of autism residing in the US and speaking English was eligible for this study. The mean age of the SPARK cohort in this analysis was 16.5 years old (SD 19.2 years). Cases made up 46% of the SPARK cohort (the remaining 54% were controls). The sex breakdown of the full SPARK cohort was 57% male and 43% female. The sex breakdown of the SPARK case cohort was 77% male and 23% female.
Recruitment	All participants were recruited by clinical sites receiving a grant from SFARI or through digital and social media or another advertisement. All recruitment materials were approved by Western IRB (http://wirb.com/) under Protocol ##20151664). All participants provided written informed consent to take part in the study. Written informed consent was obtained from all legal guardians or parents for all participants age 18 and younger and all participants age 18 and older who have a legal guardian. Assent was also obtained from dependent participants age 10 and older. The individuals with autism and/or their parents/guardians/siblings that participate in SPARK may be more interested in scientific research than other people. This should not impact the findings of the study.
Ethics oversight	All participating institutions waived IRB oversight to a central IRB, which was Western IRB, (http://wirb.com/).

Note that full information on the approval of the study protocol must also be provided in the manuscript.
